# Supplementary material for: Non‐invasive lung cancer diagnosis by detection of GATA6 and NKX2‐1 isoforms in exhaled breath condensate
Source: EMBO Mol Med. 2016 Nov 7;8(12):1380–9. doi: 10.15252/emmm.201606382 (PMC5167131; doi:10.15252/emmm.201606382)
Supplement: Supplementary file 1 — Appendix [file EMMM-8-1380-s001.pdf]

APPENDIX SUPPLEMENTARY MATERIALS

Table of contents

Appendix Supplementary Results

Appendix Supplementary Figure Legends

Appendix Figure S1: Specific PCR amplification of both isoforms of *GATA6*.

Appendix Figure S2: Specific PCR amplification of both isoforms of *NKX2-1*.

Appendix Figure S3: The use of transcript isoform expression ratios of two different genes makes the LC score robust and less sensitive to batch effects.

Appendix Table S1: Primer sequences used for the analysis of *GATA6* and *NKX2-1*.

Appendix Table S2: Values for the individual ratios of *GATA6* and *NKX2-1*, the LC score and the prediction of the LC score based classifier on the training set of samples.

Appendix Table S3: Values for the individual ratios of *GATA6* and *NKX2-1*, the LC score and the prediction of the LC score based classifier on the validation set of samples.

Appendix Table S4: Estimation of the performance metric of the LC score in a population of 100,000 current smokers using a LC prevalence of 7%.

Appendix Supplementary References.

List of guide lines for omics approaches for diagnosis purposes with clinical application: STARD Checklist, REMARK checklist and OMICS checklist.

Appendix Supplementary Results

Embryonic isoforms of GATA6 and NKX2-1 are highly expressed in human lung cancer cell lines and in several mouse models of lung cancer

Quantitative expression analysis by reverse transcription of RNA followed by polymerase chain reaction (qRT-PCR) (Fig EV2B) in human control lung tissue (Ctrl), human lung adenocarcinoma (A549, A427) and human bronchoalveolar carcinoma (H322) cell lines showed that the expression of the Em-isoforms of *GATA6* and *NKX2-1* was higher than the expression of the Ad-isoforms in the lung cancer cell lines tested. While in the human control lung tissue, we observed the opposite results, with the Ad-isoforms expressed at higher levels than the Em-isoforms. We confirmed these results in various mouse models of LC (Fig EV2C): two transgenic mouse models, in which either an oncogenic latent allele of *K-Ras* (*K-rasLA*) was activated upon spontaneous recombination (Johnson et al, 2001) or the oncogenic N-terminal deletion mutant of *Raf1* (*c-Raf-1-BxB*) was expressed in alveolar type II cells (Kerkhoff et al, 2000); a xenograft mouse model, in which human lung adenocarcinoma A549 cells were instilled intratracheally into BALB/c *nu/nu* mice (Savai et al, 2009); and a mouse model of experimental metastasis, in which mouse Lewis lung carcinoma (LLC1) cells were injected into the tail vein of C57/BL6 mice to induce tumor formation in the lung (Elkin & Vlodavsky, 2001). These animal models represent a wide array of lung tumors mimicking human disease, ranging from early stage adenomas and adenocarcinomas to metastatic tumor models. Independent of the murine model of LC analyzed, we detected elevated expression of the Em-isoforms of *Gata6* and *Nkx2-1* in all the tumors when compared to healthy lung tissue from control (Ctrl) mice. To facilitate comparability between the different LC models, we calculated the Em by Ad expression ratio (Em/Ad) for each sample. While the extent of the

increase in the Em/Ad ratio of *Gata6* and *Nxk2-1* varied among the different animal models for LC, both ratios were consistently higher in the different models as compared to the control. Therefore, our results suggest that the Em-isoforms of *GATA6* and *NKX2-1* are relevant during LC formation.

Exosomes enriched in human lung cancer contain Em- and Ad-trasncripts of GATA6 and NKX2-1

Whole cell protein extracts from control and LC tissue samples were analyzed by Western blotting using antibodies specific for the exosome markers, CD63 and TSG101 (Fig EV2D). We found increased levels of both exosome markers in protein extracts from LC samples when compared to the control samples, suggesting an enrichment of exosomes in LC tissue. For further characterization, we decided to isolate the exosomes from EBCs from control and LC patients. Isoform-specific expression analysis using RNA that was extracted from these exosomes (Fig EV2E) showed that both transcripts, Em and Ad, were present in the exosomes. Furthermore, we detected increased levels of the Em-isoform in the EBCs from LC patients that were reflected in increased Em/Ad expression ratios in these specific samples. A bacterial 16S/23S rRNA was added to the samples prior to exosome lysis as spike-in control to monitor RNA isolation and qRT-PCR efficiency.

Optimization of qRT-PCR based expression analysis in exhaled breath condensate

Following international guidelines for the development of diagnostic methods with clinical application (2012; Bossuyt et al, 2003; McShane et al, 2013a; McShane et al, 2013b), we established reproducible standard operating procedures (SOP) for a non-invasive and straightforward LC diagnosis. The SOP for EBC collection, storage and processing were optimized and consequently used in three centers by different operators. EBC is a promising

source of biomarkers for lung diseases since the condensed droplets contain a mixture of nonvolatile biomarkers such as adenosine, prostaglandins, leukotriene, cytokines, etc. and water-soluble volatile biomarkers such as nitrogen oxides (Davis et al, 2012; Effros et al, 2012; Effros et al, 2002; Horvath et al, 2005; Montuschi, 2007). EBCs are typically collected through cooling devices. Here, we tested two of the most commonly used devices for EBC collection (Jungraithmayr et al, 2008), (Carraro et al, 2010) for their suitability for subsequent RNA extraction (Fig EV3A). Using the same conditions for EBC collection and RNA extraction, the RTube showed a yield of 573±48ng RNA per 500 µl EBC (n=6), whereas the TurboDECCS showed a lower yield of 292±42ng RNA per 500µl EBC (n=6; P=0.001). Thus, we continued collecting the samples with the RTube and tested various methods of RNA extraction from EBC. A column-based method (QIAGEN RNeasy Micro kit) showed the most efficient isolation of RNA from EBC when compared to the ArrayPure RNA Purification Kit, (Epicentre) or TRIzol (Life technologies). Further, we tested different EBC volumes to determine the best starting material for RNA extraction (Fig EV3B). The RNA yield increased with the EBC volume following a sigmoid curve that reached a plateau at 573±48 ng RNA using 500 µl EBC. RNA yield did not improve significantly when more than 500µl of EBC volume was used as starting material, probably due to limitations of the method of RNA isolation used. RNA quality is critical for qRT-PCR based expression analysis (Fleige & Pfaffl, 2006). However, selection of the proper methods for RNA quantification and quality analysis is determined by the type of samples and the downstream applications after RNA purification (Shane et al, 2010; Wieczorek et al, 2012). For instance, quality assessment of RNA isolated from EBCs by the RNA integrity number (RIN) using the 2100 Bioanalyzer (Agilent Technologies) was not conclusive (data not shown), similar to results obtained for RNA purified from formalin-fixed and paraffin-embedded (FFPE) samples (Shane et al, 2010; Wieczorek et al, 2012). Nevertheless, qRT-PCR based expression of genes has been successfully performed in such samples with highly fragmented RNA due to the small size of

the amplicons detected in this downstream application (Shane et al, 2010; Wieczorek et al, 2012). Synthesis of cDNA by reverse transcription and qPCR amplification were optimized using RNA isolated with the RNeasy Micro kit from 500µl EBC collected with the RTube. Using equal amounts of starting RNA, cDNA conversion was carried out using the High Capacity cDNA reverse Transcription Kit (Applied Biosystems) and EpiScript™ Reverse Transcriptase (Epicentre). Following cDNA conversion, the expression of *GATA6 Ad* was measured in both samples (Fig EV3C). It was found that High Capacity RT Kit showed lower and reproducible C_T values (33.8±0.3), whereas Epi Script RT-PCR led to higher C_T values with higher variation among replicates (36.0±1.0). To determine whether accurate C_T values could be obtained, the starting cDNA (1:6 pre-diluted) was twofold serially diluted three times and the expression of *GATA6 Ad* was measured (Fig EV3D). We found that, C_T values obtained for the serial dilutions showed a stepwise increase of one C_T value, which would be expected of reliable measurement. In order to ensure the reproducibility of the SOP proposed here, we employed as quality criterion for the mRNA purified from EBCs the ratio of expression of the housekeeping gene *GAPDH* (gyceraldehyde 3 phosphate dehydrogenase) detected using two different primer pairs that were complementary to different regions of the mRNA (Fig EV3E, top). To increase the stringency of quality assessment for RNA isolated from EBCs, we determined similar expression ratio of a second housekeeping gene, *HPRT1* (hypoxanthine phosphoribosyltransferase 1; Fig EV3F, top). Expression ratios of *GAPDH* and *HPRT1* close to 1.0 are indicators of high integrity of mRNA (Fajardy et al, 2009). RNA purified from EBCs with expression ratios of *GAPDH* and *HPRT1* between 0.75 and 1.5 were considered as acceptable for further analysis (Fig EV3E-F, bottom). Using this mRNA quality criterion we determined that EBCs should be snap-frozen in liquid nitrogen immediately after collection because even 5 minutes incubation on ice after collection compromised mRNA quality. We also found that after long-term storage at -80°C, EBCs should be thawed on ice and further processed in less than 15 minutes (Fig EV3G). Prolonged incubation on ice or any

incubation at room temperature dramatically reduced RNA quality. We also determined that neither long term storage of EBC at -80°C nor EBC transportation on dry ice compromised the quality of RNA (Fig EV3H).

Specific PCR amplification of both isoforms of GATA6 and NKX2-1

The specificity of the different qRT-PCR products detected in the EBCs (Figs S1A-D and S2A-D) was demonstrated by dissociation curve analysis, electrophoretic gel analysis and sequencing of the different qRT-PCR products.

A minimum of 75ng of starting RNA is required for reliable isoform specific expression analysis in EBC

Serial dilution of the RNA template was used to determine the minimal material required for reliable diagnosis of LC based on the Em/Ad ratio of *GATA6* and *NKX2-1* (Fig EV4A). The expression ratio remained stable for both, control as well as LC EBC samples, until 75ng of RNA starting material. Decreasing the starting material below 75ng resulted in suboptimal detection of the Em-isoform in the control and the Ad-isoform in the LC group, which led to distorted ratios.

Repeatability of isoform-specific expression analysis in EBCs

The repeatability of isoform-specific expression analysis of *GATA6* and *NKX2-1* was confirmed by Bland-Altman plots (Bland & Altman, 1986) after measurements in two EBCs from the same patient (Test 1 and Test 2), in five patients (Fig EV4B). For *GATA6* (left panel), the mean of the differences between Test 1 and Test 2 of the five patients was 0.0155 with a 95% confidence interval (CI) from -0.004 to 0.0354 and limits of agreement between -0.028 and 0.056. Remarkably, all five differences in our experiment were within the limits of agreement. Furthermore four out of the five differences were within the 95% CI. Similar

results were obtained for *NKX2-1* (right panel), the mean of the differences between Test 1 and Test 2 of the five patients was 0.0035 with a 95% CI from 0.0006 to 0.0065 and limits of agreement between -0.003 and 0.001. All five differences were within the limits of agreement and three out of the five differences were within the 95% CI. These results confirmed the repeatability of isoform-specific expression analysis of *GATA6* and *NKX2-1* in EBCs.

Estimation of the LC score performance metrics for smoking-related lung cancer

During the external validation of our EBC-based LC diagnosis method, the performance was assessed on a population with a LC prevalence of 43.5% (Table 1, validation set of EBCs) leading to a positive predictive value (PPV) of 0.881 (Fig 2E). If we would estimate the performance metric of our LC diagnosis method on a risk group in a specific population, such as defined by (Bach et al, 2003) for smokers having at least 20 pack-years of smoking exposure, we will have to use the LC prevalence of 7%, specified for this population. Following this rationale, we have estimated the performance metric of the LC score (Appendix Table S4) when applied to a hypothetical population of 100,000 current smokers, using a LC prevalence of 7% (Bach et al, 2003), meaning that 7,000 individuals will develop LC. Since our LC score has a sensitivity of 98.3% (Fig 2E), we will detect 6,881 smokers as true positive and 119 smokers as false negative. On the other hand, there will be 93,000 smokers that will not develop LC. Based on a specificity of 89.7% (Fig 2E), we will detect 83,421 smokers as true negative and 9,579 smokers as false positives. Using these numbers, we obtain a PPV of 41.8% and a NPV of 99.9%. Although we used the sensitivity and specificity determined during the external validation of the LC score, we obtained a much lower PPV. Our observations are in accordance with previous reports (Parikh et al, 2008), the PPV is highly dependent on the prevalence of the disease.

Appendix Supplementary Figure Legends

Appendix Figure S1: Specific PCR amplification of both isoforms of *GATA6*.

A Amplification efficiency for each primer pair was calculated using serial dilutions of the cDNA template. Primer efficiency was assessed by plotting the cycle threshold values (Ct, y-axis) against the logarithm (base 10) of the fold dilution (log (Quantity), x-axis). Primer efficiency was calculated using the slope of the linear function. Data points represent mean Ct values of triplicates.

B Dissociation curve analysis of the PCR products was performed by constantly monitoring the fluorescence with increasing temperatures from 60°C to 95°C. Melt curves were generated by plotting the negative first derivative of the fluorescence (−d/dT (Fluorescence) 520nm) versus temperature (degree Celsius, °C).

C Specific PCR amplification was also demonstrated by agarose gel electrophoresis. PCR products after quantitative RT-PCR were analyzed by agarose gel electrophoresis. +, specific PCR reaction using EBC template; -, no RT control; M, 100 bp DNA ladder.

D Sequencing of the PCR products of *GATA6* Em and Ad demonstrates specific PCR amplification of both isoforms using EBC as template. Five clones for each primer pair (*GATA6* Em and Ad) were sequenced and aligned to the reference sequence (top row, yellow highlighted). Sequence similarities are represented as dots.

Appendix Figure S2: Specific PCR amplification of both isoforms of *NKX2-1*.

A Amplification efficiency for each primer pair was calculated using serial dilutions of the cDNA template. Primer efficiency was assessed by plotting the cycle threshold values (Ct, y-axis) against the logarithm (base 10) of the fold dilution (log (Quantity), x-axis). Primer efficiency was calculated using the slope of the linear function. Data points represent mean Ct values of triplicates.

B Dissociation curve analysis of the PCR products was performed by constantly monitoring the fluorescence with increasing temperatures from 60°C to 95°C. Melt curves were generated by plotting the negative first derivative of the fluorescence (−d/d*T* (Fluorescence) 520nm) versus temperature (degree Celsius, °C).

C Specific PCR amplification was also demonstrated by agarose gel electrophoresis. PCR products after quantitative RT-PCR were analyzed by agarose gel electrophoresis. +, specific PCR reaction using EBC template; -, no RT control; M, 100 bp DNA ladder.

D Sequencing of the PCR products of *NKX2-1* Em and Ad demonstrates specific PCR amplification of both isoforms using EBC as template. Five clones for each primer pair (*NKX2-1* Em and Ad) were sequenced and aligned to the reference sequence (top row, yellow highlighted). Sequence similarities are represented as dots.

Appendix Figure S3: The use of transcript isoform expression ratios of two different genes makes the LC score robust and less sensitive to batch effects. The Em/Ad ratio of *GATA6* and *NKX2-1* for controls (Ctrl) and LC EBCs in the training set (Batch 1) and the validation set (Batch 2) were log2-transformed and plotted. Boxes represent the first and third quartile, the line in the center represents the median, the error bars represent the range and the dots represent outliers.

Appendix Figure S1

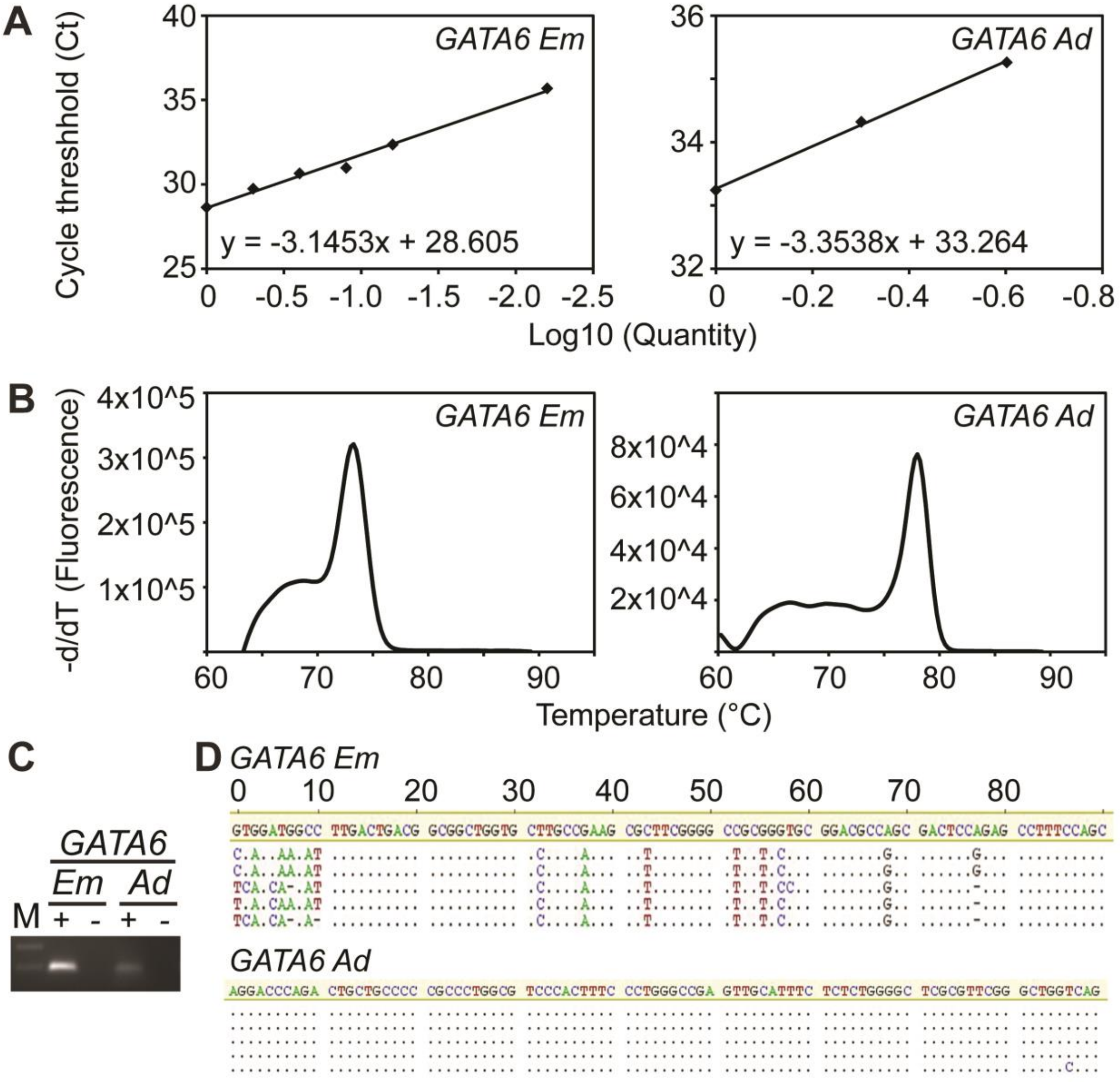

Appendix Figure S1_Mehta et al. 2016

Appendix Figure S2

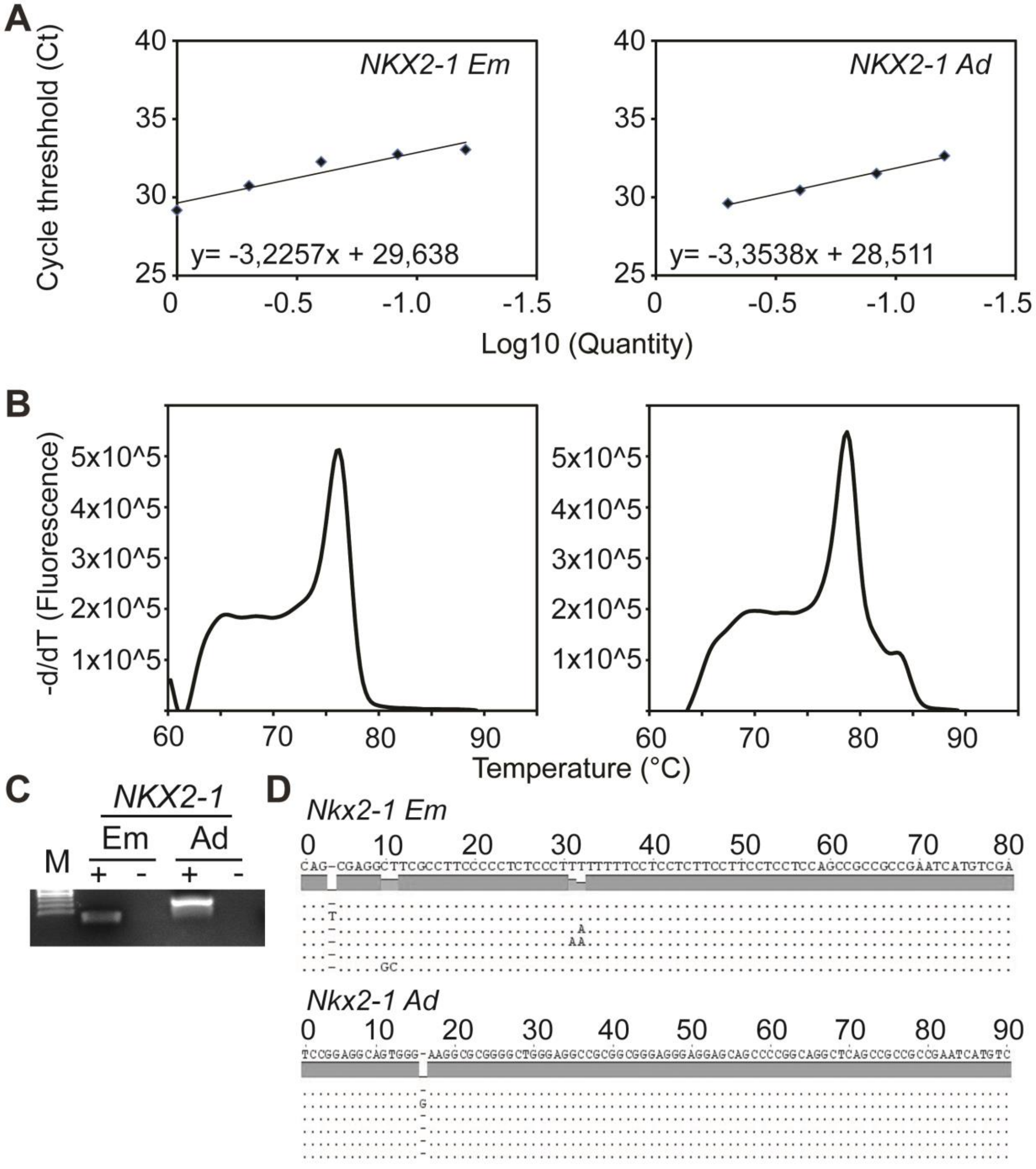

Appendix Figure S2_Mehta et al. 2016

Appendix Figure S3

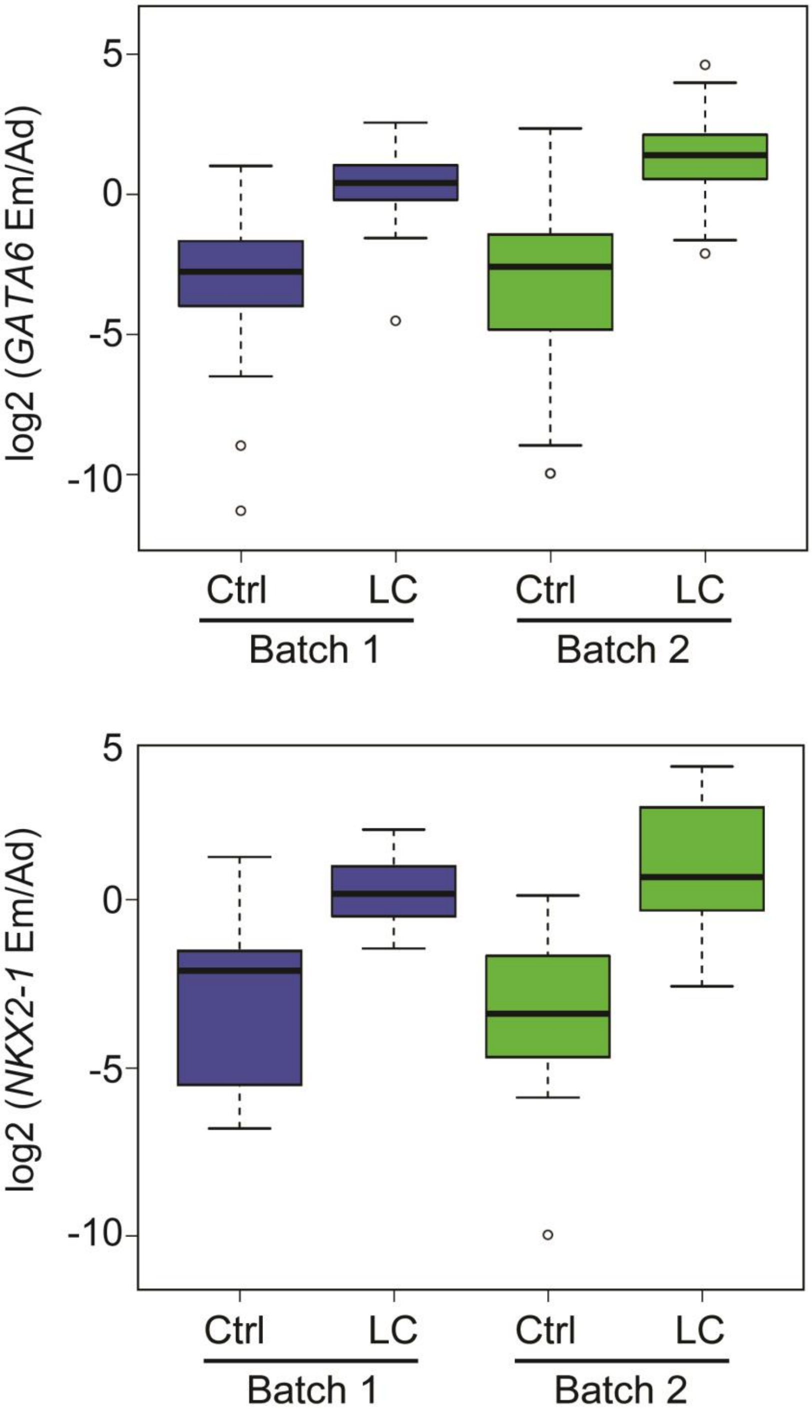

Appendix Figure S3_Mehta et al. 2016

Appendix Supplementary Tables

Appendix Table S1: Primer sequences used for the analysis of *GATA6* and *NKX2-1*.

Gene	Primer Sequence for cell lines (5'-3')	Primer Sequence for tissue, EBC (5'-3')
GATA6 Em Fwd	CTCGGCTTCTCTCCGCGCCTG	TTGACTGACGGCGGCTGGTG
GATA6 Em Rev	AGCTGAGGCGTCCCGCAGTTG	CTCCCGCGCTGGAAAGGCTC
GATA6 Ad Fwd	GCGGTTTCGTTTTCGGGGAC	AGGACCCAGACTGCTGCCCC
GATA6 Ad Rev	AAGGGATGCGAAGCGTAGGA	CTGACCAGCCCGAACGCGAG
NKX2-1 Em Fwd	AAACCTGGCGCCGGGCTAAA	CAGCGAGGCTTCGCCTTCCC
NKX2-1 Em Rev	GGAGAGGGGGAAGGCGAAGCC	TCGACATGATTCGGCGGCGG
NKX2-1 Ad Fwd	AGCGAAGCCCGATGTGGTCC	TCCGGAGGCAGTGGGAAGGC
NKX2-1 Ad Rev	CCGCCCTCCATGCCCACTTTC	GACATGATTCGGCGGCGGCT
HPRT1 5´ Fwd		TGCTGAGGATTTGGAAAGGG
HPRT1 5´ Rev		TCGAGCAAGACGTTCAGTC
HPRT1 3´ Fwd		TTTGCTTTCCTTGGTCAGGCAGT
HPRT1 3´ Rev		CGTGGGGTCCTTTTCACCAGCA
GAPDH 5´ Fwd		GGCCCGATTTCTCCTCCGGGT
GAPDH 5´ Rev		GGTGACCAGGCGCCCAATACG
GAPDH 3´ Fwd		TTCGTCATGGGTGTGAACCA
GAPDH 3´ Rev		CCAGGGGTGCTAAGCAGTTG
Gapdh Fwd		TGAGTATGTCGTGGAGTCTAC
Gapdh Rev		TGGACTGTGGTCATGAGCC
Gata6 Em Fwd		GCTAGCGCTGTTTGTTTAGGGCTCG
Gata6 Em Rev		GCCCCGAAACGCTTCGGCAG
Gata6 Ad Fwd		TTTGGGGTGGCCTCGGCTCT
Gata6 Ad Rev		CCAGGCCAACCGCACACCTT
Nkx2-1 Em Fwd		GCGGCCATGCAGCAGCAC
Nkx2-1 Em Rev		CCATGTTCTTGCTCACGTCC
Nkx2-1 Ad Fwd		ACTCTTTTGGTGGTGACTGGG
Nkx2-1 Ad Rev		CTCATGTTGCCCAGGTTGCC
16S rRNA Fwd		GATTGGTGCCTTCGGGAACT
16S rRNA Rev		AAGATAAGGGTTGCGCTCGT

Appendix Table S2: Values for the individual ratios of *GATA6*, *NKX2-1*, the LC score and the prediction of the LC score based classifier on the training set of samples.

Patient ID	Disease State	Ratio		LC score	Pred.	Correct
		GATA6	*NKX2-1*			
1	Control	0.09	0.48	-2.09	normal	yes
2	Control	0.25	0.50	-0.97	normal	yes
3	Control	0.34	0.38	-1.00	normal	yes
4	Control	0.37	0.30	-1.20	normal	yes
5	Control	0.53	0.62	0.05	LC	no
6	Control	0.09	0.23	-2.97	normal	yes
7	Control	0.15	0.41	-1.76	normal	yes
8	Control	0.11	0.32	-2.34	normal	yes
9	Control	0.26	0.55	-0.81	normal	yes
10	Control	0.28	0.24	-1.73	normal	yes
11	Control	0.47	0.29	-1.01	normal	yes
12	Control	0.10	0.31	-2.51	normal	yes
13	Control	0.31	0.34	-1.21	normal	yes
14	Control	0.26	0.19	-2.14	normal	yes
15	Control	0.45	0.24	-1.27	normal	yes
16	Control	0.24	0.13	-2.66	normal	yes
17	Control	0.15	0.44	-1.67	normal	yes
18	Control	0.22	0.25	-1.99	normal	yes
19	Control	0.30	0.31	-1.37	normal	yes
20	Control	0.29	0.34	-1.31	normal	yes
21	Control	0.27	0.21	-1.94	normal	yes
22	Control	0.11	0.30	-2.45	normal	yes
23	LC	3.20	3.58	4.09	LC	yes
24	LC	2.85	3.92	4.08	LC	yes
25	LC	1.21	2.28	2.52	LC	yes
26	LC	1.58	4.25	3.57	LC	yes
27	LC	0.82	3.58	2.68	LC	yes
28	LC	1.33	3.68	3.21	LC	yes
29	LC	1.99	2.86	3.32	LC	yes
30	LC	0.71	1.98	1.79	LC	yes
31	LC	0.87	1.74	1.85	LC	yes
32	LC	0.83	2.02	1.98	LC	yes
33	LC	2.97	1.74	3.12	LC	yes
34	LC	3.82	1.70	3.35	LC	yes
35	LC	0.04	2.41	-0.83	normal	no
36	LC	1.73	1.62	2.47	LC	yes
37	LC	3.32	2.37	3.61	LC	yes

**38**	LC	1.43	2.20	2.66	LC	yes
**39**	LC	0.78	2.10	1.97	LC	yes
**40**	LC	5.88	1.67	3.78	LC	yes
**41**	LC	2.92	0.89	2.27	LC	yes
**42**	LC	2.33	0.94	2.11	LC	yes
**43**	LC	0.34	1.84	0.94	LC	yes
**44**	LC	1.11	1.83	2.16	LC	yes
**45**	LC	0.98	1.15	1.46	LC	yes
**46**	LC	0.87	0.73	0.78	LC	yes
**47**	LC	1.12	0.94	1.35	LC	yes
**48**	LC	2.21	1.84	2.88	LC	yes
**49**	LC	0.51	0.85	0.41	LC	yes
**50**	LC	0.98	0.70	0.84	LC	yes
**51**	LC	1.68	1.12	1.99	LC	yes
**52**	LC	2.02	0.46	1.08	LC	yes
**53**	LC	1.73	0.51	1.04	LC	yes
**54**	LC	0.86	1.43	1.60	LC	yes
**55**	LC	0.98	1.14	1.46	LC	yes
**56**	LC	2.07	0.55	1.32	LC	yes
**57**	LC	1.32	0.52	0.80	LC	yes
**58**	LC	1.38	0.86	1.46	LC	yes
**59**	LC	2.37	0.37	0.97	LC	yes
**60**	LC	2.53	0.64	1.72	LC	yes
**61**	LC	0.77	0.50	0.20	LC	yes
**62**	LC	0.96	0.58	0.59	LC	yes
**63**	LC	0.80	0.94	1.00	LC	yes
**64**	LC	1.53	0.79	1.45	LC	yes
**65**	LC	1.59	0.53	1.00	LC	yes
**66**	LC	1.49	0.52	0.91	LC	yes
**67**	LC	0.70	0.56	0.24	LC	yes
**68**	LC	1.30	0.95	1.51	LC	yes
**69**	LC	1.19	0.89	1.35	LC	yes
**70**	LC	1.18	0.88	1.32	LC	yes
**71**	COPD	0.47	0.46	-0.43	normal	yes
**72**	COPD	0.81	0.58	0.42	LC	no
**73**	COPD	0.43	0.46	-0.53	normal	yes
**74**	COPD	0.39	0.49	-0.55	normal	yes
**75**	COPD	0.60	0.29	-0.75	normal	yes
**76**	COPD	1.87	0.53	1.18	LC	no
**77**	COPD	0.29	0.35	-1.26	normal	yes
**78**	COPD	2.01	2.41	3.12	LC	no
**79**	COPD	0.38	0.46	-0.63	normal	yes
**80**	COPD	0.31	0.38	-1.12	normal	yes
**81**	IPF	0.08	0.02	-6.26	normal	yes

**82**	IPF	0.16	0.02	-5.18	normal	yes
**83**	IPF	0.10	0.02	-5.81	normal	yes
**84**	IPF	0.10	0.02	-6.13	normal	yes
**85**	IPF	0.11	0.02	-5.65	normal	yes
**86**	IPF	0.09	0.02	-6.17	normal	yes
**87**	IPF	0.30	0.02	-4.90	normal	yes
**88**	IPF	0.09	0.03	-5.68	normal	yes
**89**	IPF	0.01	0.02	-8.07	normal	yes
**90**	IPF	0.01	0.30	-4.82	normal	yes
**91**	IPF	0.02	0.09	-5.50	normal	yes
**92**	IPF	0.02	0.01	-8.20	normal	yes
**93**	IPF	0.04	0.18	-4.18	normal	yes
**94**	IPF	0.09	0.03	-5.66	normal	yes
**95**	IPF	0.00	0.02	-10.29	normal	yes
**96**	IPF	0.02	0.02	-7.80	normal	yes
**97**	IPF	0.06	0.02	-6.50	normal	yes
**98**	IPF	0.03	0.12	-5.08	normal	yes
**99**	IPF	0.09	0.18	-3.34	normal	yes
**100**	IPF	0.02	0.01	-8.49	normal	yes
**101**	IPF	0.15	0.28	-2.22	normal	yes
**102**	IPF	0.04	0.02	-6.76	normal	yes
**103**	IPF	0.01	0.04	-7.45	normal	yes
**104**	IPF	0.02	0.48	-3.63	normal	yes
**105**	IPF	0.36	0.02	-4.84	normal	yes
**106**	IPF	0.04	0.04	-6.14	normal	yes
**107**	IPF	0.08	0.01	-6.63	normal	yes
**108**	IPF	0.16	0.02	-5.36	normal	yes
**109**	IPF	0.44	0.01	-4.82	normal	yes
**110**	IPF	0.04	0.03	-6.43	normal	yes
**111**	IPF	4e-04	0.16	-9.02	normal	yes
**112**	IPF	0.33	0.28	-1.40	normal	yes
**113**	IPF	0.02	0.02	-7.98	normal	yes

Appendix Table S3: Values for the individual ratios of *GATA6*, *NKX2-1*, the LC score and the prediction of the LC score based classifier on the validation set of samples.

Patient ID	Disease State	Ratio		LC Score	Pred.	Correct
		GATA6	*NKX2-1*			
**1**	LC	1.143	0.772	1.131	LC	yes
**2**	LC	13.341	9.379	6.751	LC	yes
**3**	LC	0.778	4.84	2.999	LC	yes
**4**	LC	15.75	2.55	5.316	LC	yes
**5**	LC	0.435	7.212	2.890	LC	yes
**6**	LC	1.312	9.089	4.316	LC	yes
**7**	LC	6.59	7.418	5.733	LC	yes
**8**	LC	2.121	10.778	5.023	LC	yes
**9**	LC	2.65	0.193	0.289	LC	yes
**10**	LC	0.32	7.687	2.652	LC	yes
**11**	LC	0.333	6.262	2.440	LC	yes
**12**	LC	1.706	7.88	4.412	LC	yes
**13**	LC	3.377	1.577	3.132	LC	yes
**14**	LC	2.745	9.393	5.120	LC	yes
**15**	LC	0.884	9.831	4.005	LC	yes
**16**	LC	2.472	7.884	4.795	LC	yes
**17**	LC	6.311	0.877	3.053	LC	yes
**18**	LC	24.608	0.319	3.211	LC	yes
**19**	LC	1.736	1.345	2.248	LC	yes
**20**	LC	0.777	0.409	-0.052	normal	no
**21**	LC	11.507	0.168	1.635	LC	yes
**22**	LC	3.241	4.988	4.510	LC	yes
**23**	LC	0.231	9.498	2.576	LC	yes
**24**	LC	4.921	12.917	6.116	LC	yes
**25**	LC	3.832	0.943	2.628	LC	yes
**26**	LC	1.956	1.369	2.393	LC	yes
**27**	LC	1.548	1.236	2.025	LC	yes
**28**	LC	0.446	8.236	3.080	LC	yes
**29**	LC	1.701	0.909	1.743	LC	yes
**30**	LC	3.744	6.131	4.914	LC	yes
**31**	LC	6.075	15.599	6.566	LC	yes
**32**	LC	2.832	10.042	5.234	LC	yes
**33**	LC	3.506	0.617	2.012	LC	yes
**34**	LC	6.26	1.727	3.881	LC	yes
**35**	LC	3.192	6.155	4.754	LC	yes
**36**	LC	3.033	0.627	1.882	LC	yes
**37**	LC	3.415	1.402	2.998	LC	yes

**38**	LC	1.445	2.521	2.833	LC	yes
**39**	LC	2.355	1.027	2.230	LC	yes
**40**	LC	1.59	0.71	1.368	LC	yes
**41**	LC	0.723	10.509	3.880	LC	yes
**42**	LC	4.393	3.364	4.338	LC	yes
**43**	LC	12.161	0.811	3.635	LC	yes
**44**	LC	2.449	1.744	2.924	LC	yes
**45**	LC	6.616	3.402	4.775	LC	yes
**46**	LC	10.49	0.781	3.435	LC	yes
**47**	LC	5.608	3.837	4.753	LC	yes
**48**	LC	0.416	0.793	0.120	LC	yes
**49**	LC	2.918	0.914	2.307	LC	yes
**50**	LC	0.543	3.265	2.141	LC	yes
**51**	LC	4.304	1.797	3.543	LC	yes
**52**	LC	1.937	1.142	2.159	LC	yes
**53**	LC	2.552	1.612	2.869	LC	yes
**54**	LC	2.003	1.592	2.604	LC	yes
**55**	LC	1.079	0.553	0.659	LC	yes
**56**	LC	5.078	0.844	2.781	LC	yes
**57**	LC	0.623	1.378	1.219	LC	yes
**58**	LC	1.47	1.12	1.850	LC	yes
**59**	LC	2.632	2.553	3.469	LC	yes
**60**	LC	7.759	0.736	3.050	LC	yes
**61**	Control	0.392	0.429	-0.700	normal	yes
**62**	Control	0.572	0.318	-0.679	normal	yes
**63**	Control	0.14	0.513	-1.543	normal	yes
**64**	Control	0.118	0.367	-2.133	normal	yes
**65**	Control	0.302	0.017	-4.954	normal	yes
**66**	Control	0.254	0.585	-0.765	normal	yes
**67**	COPD	0.468	0.544	-0.224	normal	yes
**68**	COPD	0.641	0.628	0.278	LC	no
**69**	COPD	0.451	0.444	-0.513	normal	yes
**70**	COPD	0.238	0.412	-1.265	normal	yes
**71**	COPD	0.179	0.144	-2.857	normal	yes
**72**	IPF	0.284	0.079	-3.121	normal	yes
**73**	IPF	0.12	0.226	-2.714	normal	yes
**74**	IPF	0.07	0.137	-3.889	normal	yes
**75**	Control	0.208	0.153	-2.627	normal	yes
**76**	Control	0.016	0.176	-5.104	normal	yes
**77**	Control	0.327	0.886	0.008	LC	no
**78**	Control	0.123	0.178	-2.983	normal	yes
**79**	Control	1.188	0.145	-0.893	normal	yes
**80**	Control	0.004	0.085	-7.435	normal	yes
**81**	Control	0.296	0.17	-2.133	normal	yes

**82**	COPD	1.44	0.218	-0.191	normal	yes
**83**	Control	0.102	0.553	-1.778	normal	yes
**84**	Control	3.535	0.798	2.338	LC	no
**85**	COPD	5.08	0.236	1.209	LC	no
**86**	Control	0.005	0.287	-5.702	normal	yes
**87**	Control	0.0138	0.258	-4.785	normal	yes
**88**	Control	2.695	0.205	0.380	LC	no
**89**	Control	0.002	0.159	-7.378	normal	yes
**90**	Control	0.001	0.315	-7.250	normal	yes
**91**	Control	0.081	0.319	-2.695	normal	yes
**92**	Control	1e-04	0.465	-9.149	normal	yes
**93**	Control	1.571	0.217	-0.107	normal	yes
**94**	Control	0.084	0.111	-3.960	normal	yes
**95**	Control	0.002	1.094	-4.998	normal	yes
**96**	Control	6.3e-06	0.108	-13.807	normal	yes
**97**	Control	0.001	0.171	-8.004	normal	yes
**98**	Control	0.165	0.203	-2.517	normal	yes
**99**	Control	3.378	0.203	0.602	LC	no
**100**	Control	0.48	0.06	-2.918	normal	yes
**101**	COPD	0.511	0.594	-0.024	normal	yes
**102**	COPD	0.435	0.565	-0.253	normal	yes
**103**	COPD	0.776	0.06	-2.422	normal	yes
**104**	IPF	0.047	0.062	-5.279	normal	yes
**105**	IPF	0.028	0.039	-6.386	normal	yes
**106**	IPF	0.013	0.054	-6.777	normal	yes
**107**	IPF	0.027	0.045	-6.247	normal	yes
**108**	IPF	0.003	0.047	-8.463	normal	yes
**109**	IPF	0.222	0.022	-4.953	normal	yes
**110**	IPF	0.192	0.027	-4.851	normal	yes
**111**	IPF	0.149	0.023	-5.310	normal	yes
**112**	IPF	0.311	0.025	-4.447	normal	yes
**113**	IPF	0.111	0.029	-5.328	normal	yes
**114**	IPF	0.035	0.058	-5.665	normal	yes
**115**	IPF	0.004	0.036	-8.495	normal	yes
**116**	IPF	0.039	0.042	-5.952	normal	yes
**117**	IPF	0.18	0.031	-4.747	normal	yes
**118**	IPF	0.165	0.033	-4.759	normal	yes
**119**	IPF	0.393	0.039	-3.657	normal	yes
**120**	IPF	0.345	0.001	-8.313	normal	yes
**121**	IPF	0.58	0.156	-1.544	normal	yes
**122**	IPF	0.008	0.072	-6.923	normal	yes
**123**	IPF	0.042	0.052	-5.612	normal	yes
**124**	IPF	0.034	0.043	-6.065	normal	yes
**125**	IPF	0.052	0.056	-5.300	normal	yes

**126**	IPF	0.39	0.06	-3.133	normal	yes
**127**	IPF	0.74	0.059	-2.492	normal	yes
**128**	IPF	0.024	0.055	-6.121	normal	yes
**129**	IPF	0.368	0.034	-3.894	normal	yes
**130**	IPF	0.04	0.028	-6.426	normal	yes
**131**	IPF	0.194	0.021	-5.150	normal	yes
**132**	IPF	0.104	0.026	-5.531	normal	yes
**133**	IPF	0.177	0.029	-4.846	normal	yes
**134**	IPF	0.361	0.027	-4.198	normal	yes
**135**	IPF	0.274	0.033	-4.235	normal	yes
**136**	IPF	0.144	0.026	-5.194	normal	yes
**137**	COPD	0.52	0.895	0.500	LC	no
**138**	COPD	0.686	0.63	0.352	LC	no

Appendix Table S4: Estimation of the performance metric of the LC score in a population of 100,000 current smokers using a LC prevalence of 7%.

**Current Smoker***	**Patients with lung cancer**†			
**EBC based diagnostic outcome**		Condition positive (7,000)	Condition negative (93,000)	
	Test outcome positive	**True positive** (TP)=6,881	**False positive** (FP)=9,579	Positive predictive value =TP/(TP+FP) =6,881/(6,881+9,579) **=41.8%**
	Test outcome negative	**False negative** (FN)=119	**True negative** (FN)=83,421	Negative predictive value =TN/(TN+FN) =83,421/(83,421+119) **=99.9%**
		Sensitivity =TP/(TP+FN) =6,881/(6,881+119) **= 98.3%**	Specificity =TN/(TN+FP) =83,421/(83,421+9,579) =**89.7%**	

*50-69 years old men and woman who had at least 20 pack-years of smoking exposure have a prevalence of 7% to develop LC (PMID:12644540). † Out of 100,000 current smokers, 7,000 will develop lung cancer.

Appendix Supplementary References

(2012) In Evolution of Translational Omics: Lessons Learned and the Path Forward, Micheel CM, Nass SJ, Omenn GS (eds). Washington (DC)

Bach PB, Kattan MW, Thornquist MD, Kris MG, Tate RC, Barnett MJ, Hsieh LJ, Begg CB (2003) Variations in lung cancer risk among smokers. Journal of the National Cancer Institute 95**:** 470-478

Bland JM, Altman DG (1986) Statistical methods for assessing agreement between two methods of clinical measurement. Lancet 1**:** 307-310

Bossuyt PM, Reitsma JB, Bruns DE, Gatsonis CA, Glasziou PP, Irwig LM, Moher D, Rennie D, de Vet HC, Lijmer JG et al (2003) The STARD statement for reporting studies of diagnostic accuracy: explanation and elaboration. Annals of internal medicine 138**:** W1-12

Carraro S, Cogo PE, Isak I, Simonato M, Corradi M, Carnielli VP, Baraldi E (2010) EIA and GC/MS analysis of 8-isoprostane in EBC of children with problematic asthma. The European respiratory journal 35**:** 1364-1369

Davis MD, Montpetit A, Hunt J (2012) Exhaled breath condensate: an overview. Immunology and allergy clinics of North America 32**:** 363-375

Effros RM, Casaburi R, Porszasz J, Morales EM, Rehan V (2012) Exhaled breath condensates: analyzing the expiratory plume. American journal of respiratory and critical care medicine 185**:** 803-804

Effros RM, Hoagland KW, Bosbous M, Castillo D, Foss B, Dunning M, Gare M, Lin W, Sun F (2002) Dilution of respiratory solutes in exhaled condensates. American journal of respiratory and critical care medicine 165**:** 663-669

Elkin M, Vlodavsky I (2001) Tail vein assay of cancer metastasis. Current protocols in cell biology / editorial board, Juan S Bonifacino [et al] Chapter 19**:** Unit 19 12

Fajardy I, Moitrot E, Vambergue A, Vandersippe-Millot M, Deruelle P, Rousseaux J (2009) Time course analysis of RNA stability in human placenta. BMC molecular biology 10**:** 21

Fleige S, Pfaffl MW (2006) RNA integrity and the effect on the real-time qRT-PCR performance. Molecular aspects of medicine 27**:** 126-139

Horvath I, Hunt J, Barnes PJ, Alving K, Antczak A, Baraldi E, Becher G, van Beurden WJ, Corradi M, Dekhuijzen R et al (2005) Exhaled breath condensate: methodological recommendations and unresolved questions. The European respiratory journal 26**:** 523-548

Johnson L, Mercer K, Greenbaum D, Bronson RT, Crowley D, Tuveson DA, Jacks T (2001) Somatic activation of the K-ras oncogene causes early onset lung cancer in mice. Nature 410**:** 1111-1116

Jungraithmayr W, Frings C, Zissel G, Prasse A, Passlick B, Stoelben E (2008) Inflammatory markers in exhaled breath condensate following lung resection for bronchial carcinoma. Respirology 13**:** 1022-1027

Kerkhoff E, Fedorov LM, Siefken R, Walter AO, Papadopoulos T, Rapp UR (2000) Lung-targeted expression of the c-Raf-1 kinase in transgenic mice exposes a novel oncogenic character of the wild-type protein. Cell growth & differentiation : the molecular biology journal of the American Association for Cancer Research 11**:** 185-190

McShane LM, Cavenagh MM, Lively TG, Eberhard DA, Bigbee WL, Williams PM, Mesirov JP, Polley MY, Kim KY, Tricoli JV et al (2013a) Criteria for the use of omics-based predictors in clinical trials. Nature 502**:** 317-320

McShane LM, Cavenagh MM, Lively TG, Eberhard DA, Bigbee WL, Williams PM, Mesirov JP, Polley MY, Kim KY, Tricoli JV et al (2013b) Criteria for the use of omics-based predictors in clinical trials: explanation and elaboration. BMC medicine 11**:** 220

Montuschi P (2007) Analysis of exhaled breath condensate in respiratory medicine: methodological aspects and potential clinical applications. Therapeutic advances in respiratory disease 1**:** 5-23

Parikh R, Mathai A, Parikh S, Chandra Sekhar G, Thomas R (2008) Understanding and using sensitivity, specificity and predictive values. Indian J Ophthalmol 56**:** 45-50

Savai R, Langheinrich AC, Schermuly RT, Pullamsetti SS, Dumitrascu R, Traupe H, Rau WS, Seeger W, Grimminger F, Banat GA (2009) Evaluation of angiogenesis using micro-computed tomography in a xenograft mouse model of lung cancer. Neoplasia 11**:** 48-56

Shane M, Kohlmeyer M, Hunter T, Tighe S (2010) RT-qPCR Analysis of Degraded RNA using Five Different Pre-Amplification Methods. Journal of Biomolecular Techniques : JBT 21

Wieczorek D, Delauriere L, Schagat T (2012) Methods of RNA Quality Assessment. Promega Corporation Web site

STARD checklist for reporting of studies of diagnostic accuracy

(version January 2003)

Section and Topic	Item #		On page #
TITLE/ABSTRACT/ KEYWORDS	1	Identify the article as a study of diagnostic accuracy (recommend MeSH heading 'sensitivity and specificity').	1-3
INTRODUCTION	2	State the research questions or study aims, such as estimating diagnostic accuracy or comparing accuracy between tests or across participant groups.	4-5
METHODS			
Participants	3	The study population: The inclusion and exclusion criteria, setting and locations where data were collected.	13-15
	4	Participant recruitment: Was recruitment based on presenting symptoms, results from previous tests, or the fact that the participants had received the index tests or the reference standard?	13-15
	5	Participant sampling: Was the study population a consecutive series of participants defined by the selection criteria in item 3 and 4? If not, specify how participants were further selected.	13-15; 5-6; Fig EV1
	6	Data collection: Was data collection planned before the index test and reference standard were performed (prospective study) or after (retrospective study)?	5-6; Fig EV1
Test methods	7	The reference standard and its rationale.	18
	8	Technical specifications of material and methods involved including how and when measurements were taken, and/or cite references for index tests and reference standard.	18 and App Supp Mat 3-6
	9	Definition of and rationale for the units, cut-offs and/or categories of the results of the index tests and the reference standard.	20; Table 1
	10	The number, training and expertise of the persons executing and reading the index tests and the reference standard.	13-15
	11	Whether or not the readers of the index tests and reference standard were blind (masked) to the results of the other test and describe any other clinical information available to the readers.	13-15; 5-6; Fig EV1
Statistical methods	12	Methods for calculating or comparing measures of diagnostic accuracy, and the statistical methods used to quantify uncertainty (e.g. 95% confidence intervals).	19-21
	13	Methods for calculating test reproducibility, if done.	Fig EV4B-C and App Supp Mat 6-7
RESULTS			
Participants	14	When study was performed, including beginning and end dates of recruitment.	13-15
	15	Clinical and demographic characteristics of the study population (at least information on age, gender, spectrum of presenting symptoms).	13-15; Tables 1 and 2
	16	The number of participants satisfying the criteria for inclusion who did or did not undergo the index tests and/or the reference standard; describe why participants failed to undergo either test (a flow diagram is strongly recommended).	5-6; Fig EV1
Test results	17	Time-interval between the index tests and the reference standard, and any treatment administered in between.	Table 2
	18	Distribution of severity of disease (define criteria) in those with the target condition; other diagnoses in participants without the target condition.	13-15; Table 2
	19	A cross tabulation of the results of the index tests (including indeterminate and missing results) by the results of the reference standard; for continuous results, the distribution of the test results by the results of the reference standard.	App Table S2 App Table S3
	20	Any adverse events from performing the index tests or the reference standard.	5-6; Fig EV1
Estimates	21	Estimates of diagnostic accuracy and measures of statistical uncertainty (e.g. 95% confidence intervals).	8-9; Figs 2D-E; Table EV4 and App Table S4

	22	How indeterminate results, missing data and outliers of the index tests were handled.	11<br>App Table S2<br>App Table S3
	23	Estimates of variability of diagnostic accuracy between subgroups of participants, readers or centers, if done.	Figs 1C-D<br>Figs 3A-C<br>Table EV1-4<br>App Fig S3
	24	Estimates of test reproducibility, if done.	Figs EV4A-C<br>and<br>App Supp Mat<br>6-7
DISCUSSION	25	Discuss the clinical applicability of the study findings.	10-13;<br>App Supp Mat 7<br>App Table S4

Guidelines for the REporting of tumor MARKer Studies (REMARK)

Section and Topic	Item #		On page #
INTRODUCTION	1	State the marker examined, the study objectives, and any prespecified hypotheses	4-5
METHODS			
Patients	2	Describe the characteristics (eg, disease stage or comorbidities) of the study patients, including their source and inclusion and exclusion criteria	13-15; Tables 1 and 2
	3	Describe treatments received and how chosen (eg, randomized or rule-based)	13-15; 5-6; Fig EV1
Specimen characteristics	4	Describe the type of biological material used (including control samples) and methods of preservation and storage	13-16; Table 1; 5-6; Fig EV1
Assay methods	5	Specify the assay method used and provide (or reference) a detailed protocol, including specific reagents or kits used, quality control procedures, reproducibility assessments, quantitation methods, and scoring and reporting protocols. Specify whether and how assays were performed blinded to the study end point.	15-19; App Supp Mat 3-7; Figs EV3 and EV4
Study design	6	State the method of case selection, including whether the study design was prospective or retrospective and whether stratification or matching (eg, by stage of disease or age) was used. Specify the time period from which cases were taken, the end of the follow-up period, and the median follow-up time.	13-15; 5-6; Fig EV1
	7	Precisely define all clinical end points examined.	13-15; 5-6; Fig EV1
	8	List all candidate variables initially examined or considered for inclusion in models.	13-15; 5-6; Fig EV1
	9	Give rationale for sample size; if the study was designed to detect a specified effect size, give the target power and effect size.	No statistical method was used to select the sample size because there was not reliable effect size estimates (differences in Em/Ad expression ratios in LC and control group). Further the SVM classifier was constructed from the data and not chosen a priori.
Statistical analysis methods	10	Specify all statistical methods, including details of any variable selection procedures and other model-building issues, how model assumptions were verified, and how missing data were handled	19-21
	11	Clarify how marker values were handled in the analyses; if relevant, describe methods used for cut point determination	19-21
RESULTS			
Data	12	Describe the flow of patients through the study, including the number of patients included in each stage of the analysis (a diagram may be helpful) and reasons for dropout. Specifically, both overall and for each subgroup extensively examined report the numbers of patients and the number of events	5-6; Fig EV1; Table 1

	13	Report distributions of basic demographic characteristics (at least age and sex), standard (disease-specific) prognostic variables, and tumor marker, including numbers of missing values.	Tables 1 and 2
Analysis and presentation	14	Show the relation of the marker to standard prognostic variables	8 and 11
	15	Present univariate analyses showing the relation between the marker and outcome, with the estimated effect (eg, hazard ratio and survival probability). Preferably provide similar analyses for all other variables being analyzed. For the effect of a tumor marker on a time-to-event outcome, a Kaplan-Meier plot is recommended.	Fig 1B;<br>Fig 2A-E;<br>Table EV1 and EV4
	16	For key multivariable analyses, report estimated effects (eg, hazard ratio) with confidence intervals for the marker and, at least for the final model, all other variables in the model	Figs 1C-D;<br>Figs 3A-C;<br>Tables EV2-3
	17	Among reported results, provide estimated effects with confidence intervals from an analysis in which the marker and standard prognostic variables are included, regardless of their statistical significance	App Supp Mat 7<br>App Table S4
	18	If done, report results of further investigations, such as checking assumptions, sensitivity analyses, and internal validation.	11;<br>App Supp Mat 7<br>App Table S4
DISCUSSION	19	Interpret the results in the context of the prespecified hypotheses and other relevant studies; include a discussion of limitations of the study.	9-12
	20	Discuss implications for future research and clinical value	12

Supplementary Materials:

OMICS guidelines

Specimen issues

1. Establish methods for specimen collection and processing and appropriate storage conditions to ensure the suitability of specimens for use with the omics test.
   In order to minimize variability of the diagnostic assay every step in the 'life cycle' of the biological specimen was taken into consideration.

1.1 At the first step the condition of the host or participant was considered. EBC collection was performed using the RTube (Respiratory Research) as described online (http://www.respiratoryresearch.com/products-rtube-how.htm). For sample (EBC) collection, it was recommended that all participants refrain from eating and drinks (except water) for 2 hours before collection. Participants were awake and breathing normally without mechanical ventilation. Prior to EBC collection participants were asked to rinse the mouth with fresh water to avoid any additional contaminants. Sample was collected with the Rtube using a nose clamp to avoid nasal contaminants and breathing was only through the mouthpiece.

1.2 For each participant, EBC collection was performed for 10 minutes of tidal breathing. However, if the participants felt any discomfort and/or inability to continue, a minimum time of 5 minutes was acceptable without any loss in quality of the material obtained.

1.3 After EBC collection, the samples were stored immediately at -80°C in 500µl aliquots. It is essential that the samples are frozen as soon as possible after EBC collection (see FigureEV3E-F). The EBC was stored in microcentrifuge tubes that were treated with Rnase Zap (Life technologies) and autoclaved twice. All steps during the collection and processing of EBCs were performed under RNase-free conditions, including the use of barrier-filter tips and cleaning all surfaces and gloves with Rnase Zap, which are critical to ensure the integrity and quality of the samples.

2. Establish criteria for screening out inadequate or poor-quality specimens or analytes isolated from those specimens before performing assays.
   In order to screen out inadequate or poor quality specimen, the protocol was evaluated at three key steps.

2.1 The duration of EBC collection is critical. In order to obtain sufficient material, following the guidelines for EBC sampling by the ERS/ATS Task Force a collection time of 10 minutes was used [PMID: 16135737]. All participants were requested to breathe for 10 minutes. In the case that participants experienced significant discomfort, cough and/or an inability to continue, samples in which the participants breathed for less than 5 minutes were discarded. In most published studies, a collection time of 10 minutes has been used which is recommended for two reasons,

as it results in 1-2ml of condensates from adults and subjects can usually tolerate this sampling time without fatigue and/or discomfort [PMID: 16135737].

2.2 Keeping in mind the thermolabile nature of RNA, as a second precaution, the temperature of the cooling sleeve during condensation was considered critical [PMID: 16135737]. Condensation can be achieved at temperature ≤ 0°C, and EBC can be collected as a fluid. The cooling sleeve was cooled in a frost free -20°C freezer for at least 30 minutes prior to collection. However, it has been noted that the collecting surface warms up from the exhaled air and this influences the collecting temperature. For the samples wherein the cooling sleeve was found to be insufficiently cool (temperature > 0°C), resulting in inefficient condensation, were considered to be of poor quality and were excluded.

2.3 Finally, mRNA quality was evaluated by the comparative expression of two housekeeping genes, *TUBA1A* and *HPRT1*. For both genes, PCR amplification was performed in triplicates. CT values up to 35 cycles were considered to be of good quality and included in the analysis. Furthermore, for the samples wherein the quantity of starting material was low, CT values between 35 and 38 were obtained. However, in these cases the variability between technical replicates was taken into consideration, such that samples in which the CT values exceeded 35 but the standard deviation was <1.0 were included in the analysis.

3. Specify the minimum amount of specimen required.

The minimum amount of specimen was evaluated at all stages of assay development:

3.1 EBC Collection was carried out at different time intervals, starting at 2, 5, 7 and 10 minutes. A minimum time for EBC Collection was established at 5 min such that approximately 500µl of EBC was obtained. EBC Collection when performed for 2 minutes resulted in 150-250µl of EBC.

Time duration (min)	EBC Volume (µl)
2	150±88
5	490±150
7	1000±250
10	1500±300

3.2 Total RNA isolation with the RNeasy Micro kit was performed using 200, 350, 500 or 1000µl of EBC as starting material. A minimum volume of 200µl of EBC was found to be the sufficient for RNA isolation, however, 500µl was considered optimal. (Figure EV3B).

3.3 Further, serial dilution of the RNA template was used to determine the minimal material required for reliable diagnosis of LC based on the Em/Ad ratio of *GATA6*

and *NKX2-1* (Figure EV4A). The expression ratio remained stable for both, control as well as LC EBC samples, until 75ng of RNA starting material. Decreasing the starting material below 75ng resulted in suboptimal detection of the Em-isoform in the control and the Ad-isoform in the LC group, which led to distorted ratios.

4. Determine the feasibility of obtaining specimens that will yield the quantity and quality of isolated cells or analytes needed for successful assay performance in clinical settings.
   Obtaining EBC specimen from patients is straightforward, feasible, fast and non-invasive. The EBC collection device is portable and the collection can be performed at the patient's bed side. This non-invasive handheld device is fully self-contained and disposable, ensuring sterile sample collection without cross contamination between patients. After collection, the condensate is collected using the provided plunger apparatus, eliminating the need for large laboratorial equipments like a centrifuge.

   To evaluate the feasibility of collecting the needed quantity and quality of specimens in all the clinical settings across this multicenter analysis, a preliminary feasibility assay was performed to identify the critical parameters for all steps. During the preliminary feasibility assay, EBC collection was optimized at one center (Germany) with respect to sample collection, RNA isolation, cDNA synthesis, sample storage and subsequent transportation:

5.1 Here, we tested two of the most commonly used devices for EBC collection [18764914, 19897556] for their suitability for subsequent RNA extraction (Figure EV3A). Using the same conditions for EBC collection and RNA extraction, the RTube showed a yield of 573±48ng RNA per 500 µl EBC (n=6), whereas the TurboDECCS showed a lower yield of 292±42ng RNA per 500µl EBC (n=6; P=0.001). Thus, we continued collecting the samples with the RTube and tested various methods of RNA extraction from EBC.

5.2 A column-based method (QIAGEN RNeasy Micro kit) showed the most efficient isolation of RNA from EBC when compared to the ArrayPure RNA Purification Kit, (Epicentre) or TRIzol (Life technologies) (data not shown).

5.3 Further, we tested different EBC volumes to determine the best starting material for RNA extraction (Figure EV3B). Following isolation, RNA concentrations were estimated by using a micro volume UV-Vis spectrometer (NanoDrop 2000, Thermo Scientific). For quantification of RNA, the absorbance at 260 nm and 280 nm of 1.2µl of the eluate was measured. For concentration estimation, the optical density (OD) is measured by the spectrometer, where the OD is directly proportional to the concentration of RNA, such that, $1\ \mathrm{OD}_{260} = \frac{40\mathrm{ng}}{(\mu\mathrm{l})}$ of RNA. The RNA yield increased with the EBC volume following a sigmoid curve that reached a plateau at 573±48 ng RNA using 500 µl EBC. However, the RNA yield did not improve significantly when more than 500µl of EBC volume was used as starting material, probably due to a limitation of the method of RNA isolation used.

5.4 Synthesis of cDNA by reverse transcription and qPCR amplification were optimized using RNA isolated with the RNeasy Micro kit from 500µl EBC collected with the RTube (FigureEV3C/D).

5.5 Since this was planned and executed as a multicenter trial, immediate sample processing was not possible and long term storage and transport of samples was considered relevant. We employed as quality criterion for the mRNA purified from EBCs the ratio of expression of the housekeeping gene *GAPDH* (gyceraldehyde 3 phosphate dehydrogenase) detected using two different primer pairs that were complementary to different regions of the mRNA (Figure EV3E, top). To increase the stringency of quality assessment for RNA isolated from EBCs, we determined similar expression ratio of a second gene that is also commonly used as internal control for qRT-PCR based expression analysis, *HPRT1* (hypoxanthine phosphoribosyltransferase 1; Figure EV3F, top). Expression ratios of *GAPDH* and *HPRT1* close to 1.0 are indicators of high integrity of mRNA [19284566]. RNA purified from EBCs with expression ratios of *GAPDH* and *HPRT1* between 0.75 and 1.5 were considered as acceptable for further analysis.

   a. Using this mRNA quality criterion we determined that EBCs should be snap-frozen in liquid nitrogen immediately after collection because even 5 minutes incubation on ice after collection compromised mRNA quality (Figure EV3E-F, bottom).

   b. We also found that after long term storage at -80°C, EBCs should be thawed on ice and further processed in less than 15 minutes (Figure EV3G). Prolonged incubation on ice or any incubation at room temperature dramatically reduced RNA quality.

   c. We also determined that neither long term storage of EBC at -80°C (upto 1 year) nor EBC transportation on dry ice compromised or influenced the quality of RNA (Figure EV3H).

Assay issues

5. Review all available information about the standard operating procedures (SOPs) used by the laboratories that performed the omics assays in the developmental studies, including information on technical protocol, reagents, analytical platform, assay scoring, and reporting method, to evaluate the comparability of the current assay to earlier versions and to establish the point at which all aspects of the omics test were definitively locked down for final validation.

5.1 In order to obtain sufficient material, a recommended collection time of 10 minutes was used [PMID: 16135737]. All participants were requested to breathe for 10 minutes. In the case that participants experienced significant discomfort, cough and/or an inability to continue, samples in which the participants breathed for less than 5 minutes were discarded. In most published studies, a collection time of 10 minutes has been used which is recommended for two reasons, as it results in 1-2ml

of condensates from adults and subjects can usually tolerate this sampling time without fatigue and/or discomfort [PMID: 16135737].

5.2 Two of the most commonly used devices for EBC collection [18764914, 19897556] were tested for their suitability for subsequent RNA extraction (Figure EV3A). Using the same conditions for EBC collection and RNA extraction, the RTube showed a yield of 573±48ng RNA per 500 µl EBC (n=6), whereas the TurboDECCS showed a lower yield of 292±42ng RNA per 500µl EBC (n=6; P=0.001). Thus, we continued collecting the samples with the RTube and tested various methods of RNA extraction from EBC.

5.3 A column-based method (QIAGEN RNeasy Micro kit) showed the most efficient isolation of RNA from EBC when compared to the ArrayPure RNA Purification Kit, (Epicentre) or TRIzol (Life technologies) (data not shown).

5.4 Further, we tested different EBC volumes to determine the best starting material for RNA extraction (Figure EV3B). Following isolation, RNA concentrations were estimated by using a micro volume UV-Vis spectrometer (NanoDrop 2000, Thermo Scientific). For quantification of RNA, the absorbance at 260 nm and 280 nm of 1.2µl of the eluate was measured. For concentration estimation, the optical density (OD) is measured by the spectrometer, where the OD is directly proportional to the concentration of RNA, such that, $1\ \mathrm{OD}_{260} = \frac{40\mathrm{ng}}{(\mu\mathrm{l})}$ of RNA. The RNA yield increased with the EBC volume following a sigmoid curve that reached a plateau at 573±48 ng RNA using 500 µl EBC. RNA yield did not improve significantly when more than 500µl of EBC volume was used as starting material, probably due to a limitation of the method of RNA isolation used.

5.5 Synthesis of cDNA by reverse transcription and qPCR amplification were optimized using RNA isolated with the RNeasy Micro kit from 500µl EBC collected with the RTube (FigureEV3C).

5.6 Keeping in mind the rapidly evolving technologies, two gene expression assays were selected for analysis due to their sensitivity for low copy detection: SYBR Green based quantitative PCR and TaqMan based PCR. Plasmids containing the cloned PCR-product are commonly used as standards for absolute quantification and normalization of specific gene expression. To determine the possible detection range of both assays, plasmid standards were serially diluted from 106 copies to 1 copy. For both SYBR green and TaqMan gene expression assay, the same primer sets were used with the addition of hybridization probes for TaqMan assay. Sensitivity of both gene expression assays was validated by fitting the data points

with a linear regression analysis $(f) = mx + b$, where m is the slope of the line, b the intercept of the y-axis and x the independent variable of the function f(x). Using the same plasmid standard in both TaqMan and SYBR green chemistry, the results showed that both assays show detect the targets even in a single copy reaction.

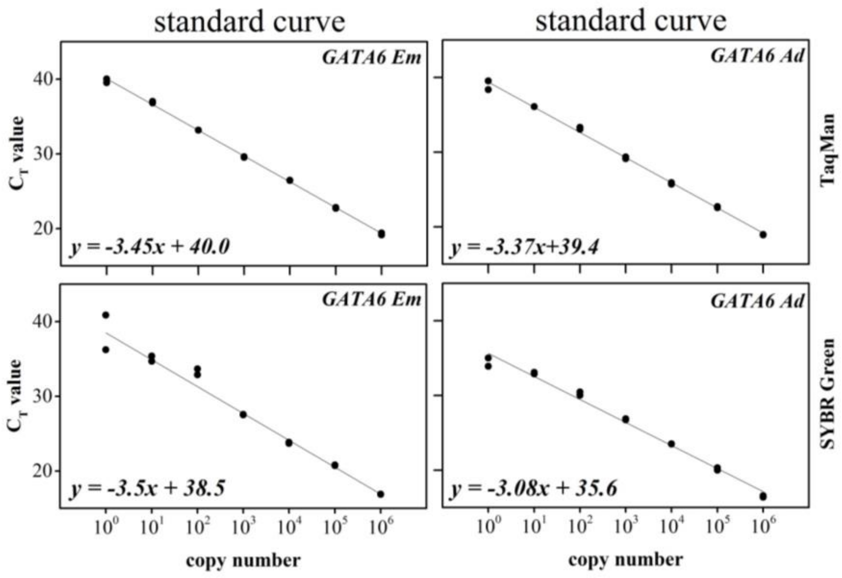

Figure 1: Sensitivity of TaqMan and SYBR Green based gene expression assay for *GATA6* Em and Ad. Serial dilutions of plasmid template (10^6 to 1 copy) was used to analyze the sensitivity of both SYBR Green and TaqMan based assay for low copy detection. CT values were plotted against the copy number and linear regression was calculated as y = mx + b. Dots represent mean CT value of triplicates.

5.6 Further, serial dilution of the RNA template was used to determine the minimal material required for reliable diagnosis of LC based on the Em/Ad ratio of *GATA6* and *NKX2-1* (Figure EV4A). The expression ratio remained stable for both, control as well as LC EBC samples, until 75ng of RNA starting material. Decreasing the starting material below 75ng resulted in suboptimal detection of the Em-isoform in the control and the Ad-isoform in the LC group, which led to distorted ratios.

The assay was locked down to perform under the following conditions:

Step	Optimal conditions	Minimal conditions
EBC Collection	10 minutes	5 minutes
EBC Processing	Aliquots of 500µl, using barrier filter tips<br>Snap freezing immediately using liquid nitrogen/dry ice	
EBC Storage	-80°C	
EBC Transportation	Dry ice	
RNA Isolation	Starting material: 500µl EBC<br>Elution volume: 12µl	Starting material: 200µl EBC<br>Elution volume: 12µl
cDNA synthesis	500-700ng	75ng

qRT-PCR	1x concentration of the SYBR® Green master mix, 250nM each forward and reverse primer and as template 3.5µl from a 6 fold diluted RT reaction were used for the gene specific qPCR reaction.	

The locked down assay requires the following instruments and reagents:

Step	Product Name	Company Name
**EBC Collection**	Rtube	Respiratory Research
**EBC Processing**	Barrier Filter Tips (1ml)	Biozym
**RNA Isolation**	Rneasy Micro Kit	Qiagen
**RNA Quantification**	NanoDrop2000	Thermo Scientific
**cDNA synthesis**	High Capacity cDNA Reverse Transcription Kit	Applied Biosystems
**qRT-PCR**	Power SYBR® Green PCR Master Mix	Applied Biosystems
	Step One Plus Real-Time PCR System	Applied Biosystems

The most critical point to ensure the needed quantity and quality of the specimens is to maintain RNase free conditions. Specifically, all surfaces, pipettes, tubes and the gloves of the handler should be cleaned with RNase Zap (Ambion) to avoid RNase contamination. All tubes were cleaned with RNase Zap and autoclaved two times to ensure RNase free conditions. Further, barrier-filter tips were used during all steps.

This standardized, optimized protocol was used in all three centers. The assay was identical in all three centers with respect to the collection device, EBC collection protocol, and storage conditions. RNA isolation and expression analysis was performed in one centralized center with identical working conditions for all samples.

6. Establish a detailed SOP to conduct the assay, including technical protocol, instrumentation, reagents, scoring and reporting methods, calibrators and analytical standards, and controls.

In order to minimize variation in the result when the assay is performed at different times, in different laboratories, and by different operators, the assay was locked-down with the following conditions:

6.1. SAMPLE COLLECTION AND PROCESSING

1. EBC Collection was performed using the Rtube, Respiratory Research.
2. All tubes, pipettes and gloves were cleaned using RNase Zap (Ambion) to minimize RNase contamination.
3. Barrier-filter tips were used to transfer the condensate to RNase free-microcentrifuge tubes (Eppendorf).
4. Time duration of EBC Collection was established at 10 minutes of tidal breathing. A minimum time of 5 minutes EBC Collection time is acceptable.
5. Following EBC Collection, samples were snap frozen in aliquots of 500µl as soon as possible to avoid sample degradation.

6.2. RNA ISOLATION, cDNA SYNTHESIS AND qRT-PCR

6. RNA Isolation was performed using 500µl EBC starting material using the Rneasy Micro RNA Isolation kit (Qiagen), with an elution volume of 12µl Rnase free water.
7. cDNA Synthesis was carried out using the High capacity cDNA Synthesis kit (Applied Biosystems) using a minimum of 75ng RNA as starting material.
8. Resulting cDNA was diluted 1:6 and qRT-PCR analysis was performed using the Power SYBR Green Mix (Applied Biosystems) on the StepOne Plus system. For qRT-PCR, a reaction volume of 10 µl was taken and 3.5 µl of the diluted cDNA was used per reaction. Each reaction was performed in triplicates.
9. For every sample, qRT-PCR was performed in triplicates, using primers specific for *TUBA1A*, *HPRT1*, *GATA6* Em, *GATA6* Ad, *NKX2-1* Em and *NKX2-1* Ad.
10. Following PCR amplification, mRNA quality was first evaluated by the comparative expression of two housekeeping genes, *TUBA1A* and *HPRT*. For both genes, PCR amplification was performed in triplicates. CT values up to 35 cycles were considered to be of good quality and included in the analysis. Further, for the samples wherein the quantity of starting material was low, CT values between 35 and 38 were obtained. However, in these cases the varibility between technical replicates was taken into consideration. Samples in which the CT values exceeded 35 but the standard deviation was <1.0 were included in the analysis. Samples that exhibited higher CT values or greater variability were excluded.

6.3. CONTROLS

11. As the ‘calibrator’, serial dilutions of the plasmids containing the cloned PCR-product were used. Plasmid standards were serially diluted from 10^3 copies to 1 copy, which would generate a specific CT value for each run.
12. As the ‘analytical standard’, cDNA from Human lung cancer cell lines, A549 and H520 was used. These samples have been extensively analyzed and gave unequivocally positive results.

13. As a ‘control’, cDNA from a control donor lung tissue was used, as the sample that would give unequivocally negative results. As the control that provides unequivocally positive results, cDNA from human lung tumor biopsies were used.

6.4. CALCULATION OF EXPRESSION RATIOS AND LC SCORE

14. Following qRT-PCR, the Em/Ad ratios of GATA6 and NKX2-1 were calculated according to the 2-ΔΔCT method.
15. After calculating the ratios, the LC score was calculated using the formula:

$$\text{LC Score} = 0.715 \times \log_2\left(\frac{\text{GATA6 Em}}{\text{GATA6 Ad}}\right) + \times \log_2\left(\frac{\text{NKX2} - 1\text{ Em}}{\text{NKX2} - 1\text{ Ad}}\right) \text{x } 0.855 + 1.3122$$

7. Establish acceptability criteria for the quality of assay batches and for results from individual specimens.

In order to ensure acceptable quality between assay batches, the following steps were carried out:

1. Control samples (Control donor and lung tumor biopsies) and the analytical standards (A549 cDNA) were used consistently in all assays.
2. To avoid artifacts from unexpected technical problems, including differences in batches of consumable reagents, randomly chosen EBC samples were reanalyzed thereby detecting any assay problems and ensuring consistency of results.
3. Lastly, each individual sample was measured in triplicates; therefore making it possible to identify unreliable readings.

8. Validate assay performance by using established analytical metrics such as accuracy, precision, coefficient of variation, sensitivity, specificity, linear range, limit of detection, and limit of quantification, as applicable.

For the described assay, two pairs of biomarkers (*GATA6* and *NKX2-1*) were measured in control and lung cancer samples. These two pairs of biomarkers resulted in two independent ratios (Em/Ad) that were used to predict lung cancer among patients. Further, the two individual biomarker measurements were combined to yield a binary clinical score, called the Lung Cancer Score (LC Score). The analytical performance characteristics of the individual biomarker measurements as well as that of the LC Score were evaluated with respect to the accuracy, sensitivity and specificity using 10 fold cross validation as well as bootstrapping. For translation to a clinical classification, a cut off point of 0 was used for the LC Score.

	**GATA6**	**NKX2-1**	**LC Score**
Area Under ROC Curve	0.93	0.96	0.99

External validation of the LC score classifier was performed on an independent set of samples (EBCs) consisting of 138 previously unseen samples (78 controls and 60 LC patient EBCs; Table 1; Figure 2C). These EBCs were collected mimicking conditions of clinical use, for instances they were collected in different centers by different operators according to the final optimized SOP. Performance assessment of the LC score classifier by applying it to the independent validation set of EBCs (Figure 2E) confirmed its high performance by achieving sensitivity of 98.3%, and a specificity of 89.7%.

9. Establish acceptable reproducibility among technicians and participating laboratories and develop a quality assurance plan to ensure adherence to a detailed SOP and maintain reproducibility of test results during the clinical trial.

Following international guidelines, we established reliable and reproducible SOP for the different steps comprising our method of LC diagnosis (Figure S1) such as sample collection, storage and processing; assay operation, reproducibility, reliability and feasibility for clinical use; predictor model development and validation as well as assessment of overall method performance using established analytical metrics as accuracy, sensitivity and specificity. RNA isolation and qRT-PCR was performed in one centralized center thereby reducing variability due to technical differences arising from instrumentation and/or platforms. The initial assay in the training set was performed by two independent individuals and was later confirmed in the test set of samples.

In order to evaluate the quality of the assay, calibrator and analytical standards were used in the assays to determine the differences among operators, avalaible instrumentation and/or batches of consumable reagents used. These samples were known to produce specific values for the assay therefore providing quality assurance between the different runs. The expected values for the samples are below.

Copy Number	CALIBRATORS			
	GATA6 Em		*GATA6* Ad	
	CT Mean	CT Stdev	CT Mean	CT Stdev
1000000	16.869705	0.01776959	16.470105	0.13975966
100000	20.753885	0.04711452	20.155595	0.22297198
10000	23.749375	0.10642664	23.49907	0.01110158
1000	27.54239	0.04552353	26.79374	0.10356286
100	33.265775	0.53903457	30.220555	0.30802278
10	35.02983	0.48377418	32.984505	0.13891113
1	38.549565	3.26870716	34.445085	0.76822202

Sample	CONTROLS			
	GATA6 Em		*GATA6* Ad	
	CT Mean	CT Stdev	CT Mean	CT Stdev
A59	28.0073333	0.116423079	32.01019757	0.384956435

H520	28.84463333	0.064565496	31.241	0.372943695

10. Establish a turnaround time for test results that is within acceptable limits for use in real-time clinical settings.

Using the optimized conditions the turnaround time of the current assay is 6 hours. However, the assay has a high throughput and can be performed for up to 6 individuals simultaneously.

Model development, specification, and preliminary performance evaluation

11. Evaluate data used in developing and validating the predictor model to check for accuracy, completeness, and outliers. Perform retrospective verification of the data quality if necessary.

    There is one LC sample with an extraordinary low GATA6 ratio of 0.044 (Patient ID35, Appendix Table S2) in the training set. There are additional control samples (for example, patient IDs 71, 76, 78, Appendix Table S2) which have high *GATA6* and *NKX2-1* ratios. However, these are all samples of COPD patients which are known to have a higher risk to develop lung cancer. These samples have a measurable influence on the construction of the SVM classifier in the training set, since it lies close to the decision boundary (Figure 2B). There is another control sample which shows an extraordinary low *NKX2-1* ratio of 0.01 (patient ID 92, Appendix Table S2). This sample however is far from the decision boundary in any case and does not influence the construction of the SVM classifier. Moreover, note that these extreme ratios are not as extreme as it seems at first sight, since similar ratios (and even more extreme ratios) are regularly observed for the IPF samples.

    In the validation set, there is one LC sample with an extraordinarily low ratio of both *GATA6* and *NKX2-1* (Patient ID: 20, Appendix Table S3) which was misclassified and was a false negative. This sample has been undergoing active treatment for lung cancer, which may be a plausible explanation for the reduced ratios. As in the training set, two COPD samples showed a higher ratio for *GATA6* and *NKX2-1* and were misclassified as LC (Patient IDs 68, 85, Appendix Table S3). However, none of the measured values need to be considered as an outlier, when the entire study population is taken into consideration.

12. Assess the developmental data sets for technical artefacts (for example, effects of assay batch, specimen handling, assay instrument or platform, reagent, or operator), focusing particular attention on whether any artefacts could potentially influence the observed association between the omics profiles and clinical outcomes.

    One of the strengths of our approach is that batch effects are unlikely; because it is based on the measurement of Em/Ad transcript isoform ratios which are insensitive to typical batch effects like sample preparation and RNA extraction efficiency. Also, primer-associated amplification biases will alter all ratios by the same factor. Since our SVM classifier is linear (on the log scale), such biases do not affect classification accuracy, making the LC score robust and less sensitive to batch effects. We compared the LC scores of LC and control samples in Batch1 vs. Batch2 with

respect to their *GATA6* and *NKX2-1* isoform ratios (Appendix Figure S3). Although a slight batch effect is visible, the cancer state (LC or control) has a much stronger effect.

13. Evaluate the appropriateness of the statistical methods used to build the predictor model and to assess its performance.
    The linear support vector machine (SVM) is a standard classification algorithm. We preferred SVM over other common methods like linear Discriminant analysis (LDA, because LDA relies on the assumption of normally distributed data, which does not hold in our case), k-nearest neighbors (kNN, this leads to a classifier which is less easy to interpret, and kNN does not give rise to a score like our LC score) or logistic regression (this also gives linear decision boundaries, however it is sensitive to extreme samples/values).
    The performance of the SVM was assessed twofold: First, an initial SVM was learned on a training set, and its accuracy/sensitivity/specificity was then assessed on an independent test set from a different batch of samples. Since both sensitivity and specificity are high, this also indicates the lack of batch effects. After having demonstrated the predictive power of the SVM, the whole data set was used for the construction of the improved LC score/classifier to be used in future applications. The performance of the improved classifier was assessed via 10-fold cross-validation.
