# Supplementary material for: Non‐invasive lung cancer diagnosis by detection of GATA6 and NKX2‐1 isoforms in exhaled breath condensate
Source: EMBO Mol Med. 2016 Nov 7;8(12):1380–9. doi: 10.15252/emmm.201606382 (PMC5167131; doi:10.15252/emmm.201606382)
Supplement: Supplementary file 2 — Expanded View Figures PDF [file EMMM-8-1380-s002.pdf]

Expanded View Figures

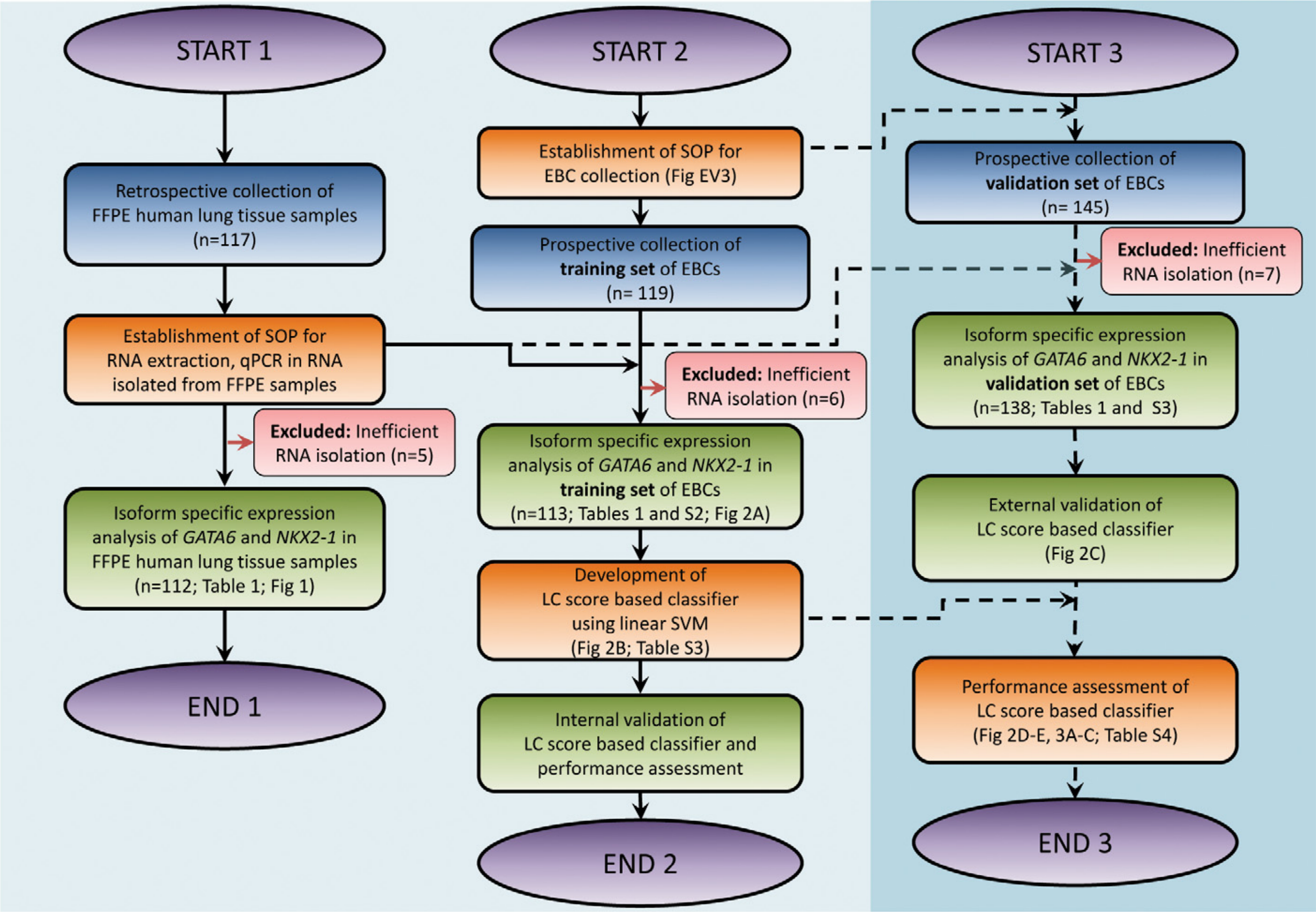

Figure EV1. Flowchart with the different steps during development of the EBC-based LC diagnostic method.

The study was started in three phases. At the first phase (START 1 to END1), formalin-fixed and paraffin-embedded (FFPE) human lung tissue samples were retrospectively collected in three centers in Germany (GER) and Mexico (MEX). The standard operation procedures (SOP) for the assay consisting of isoform-specific expression analysis of *GATA6* and *NKX2-1* were established on these FFPE samples at one center to avoid variability due to technical differences arising from instrumentation and/or platforms. Only five samples were excluded due to inefficient RNA isolation. The FFPE samples were analyzed by different operators using the optimized SOP for the assay. In the second phase (START 2 to END2), the SOP for collection, storage, and processing of exhaled breath condensates (EBC) were established at one center. The optimized SOP for EBC handling was used at three centers by different operators for the collection of the training set of samples consisting of EBCs and the corresponding FFPE samples from prospectively enrolled controls and patients with lung cancer (LC). Isoform-specific expression of *GATA6* and *NKX2-1* was analyzed on the training set using the optimized SOP for the assay. Further, a linear support vector machine (SVM) classifier was used to combine the Em/Ad ratios of *GATA6* and *NKX2-1* of each sample to create the LC score. At the third phase (START 3 to END3), an independent set of EBCs (validation set) consisting of 145 previously unseen samples were prospectively collected by different operators at different centers without prior knowledge of the clinical diagnosis (blinded sample collection) mimicking conditions of clinical use. Seven samples were excluded due to inefficient RNA isolation. The training and validation sets were comparable in their distribution of controls and LC samples, smoking history, age, and gender of the individuals (Tables 1 and 2). The LC score-based classifier was applied to the validation set of EBCs for external validation and achieved high performance. Detailed information of the study population can be found in the Material and Methods section and Tables 1 and 2.

© 2016 The Authors

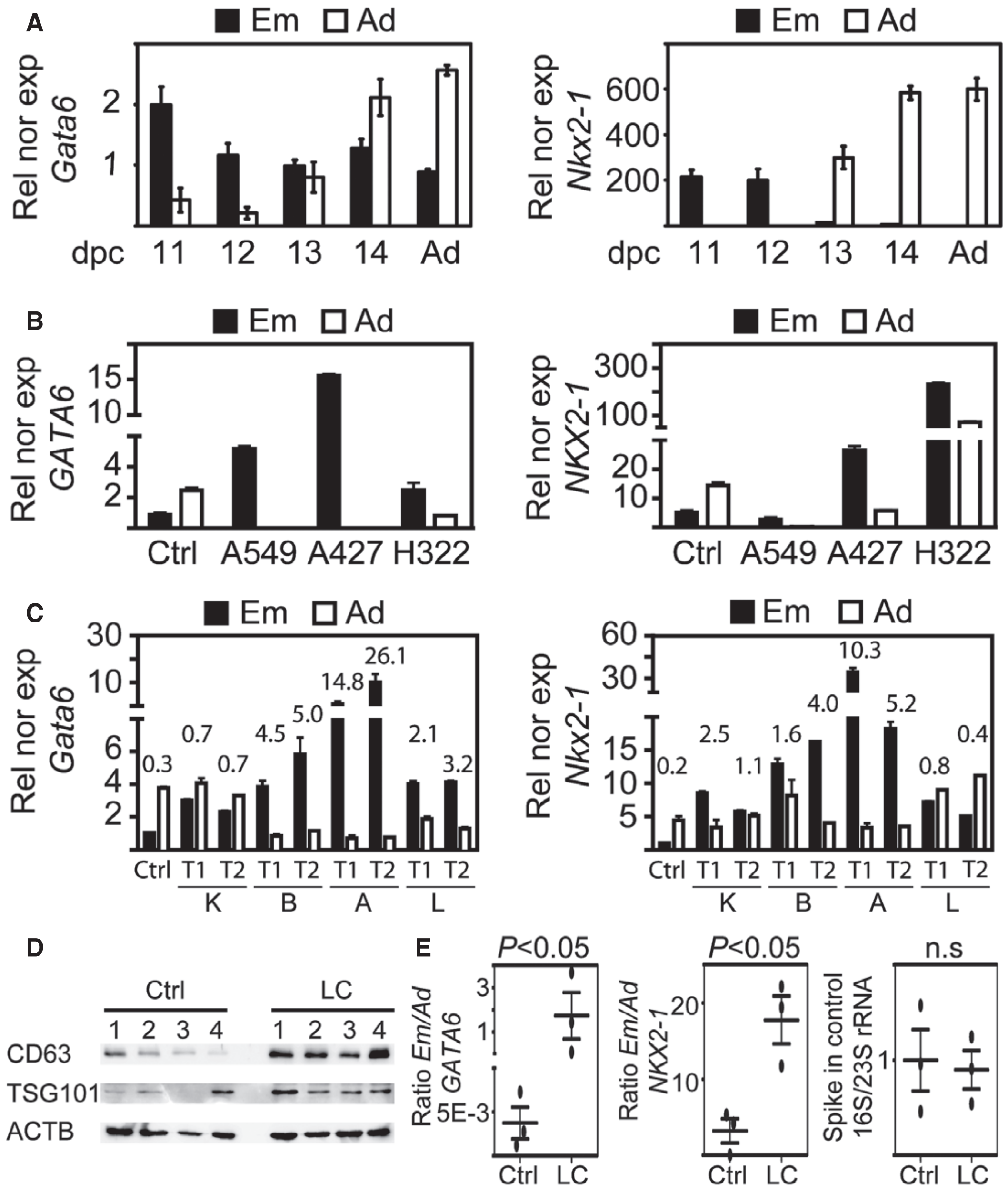

Figure EV2. Embryonic isoforms of *GATA6* and *NKX2-1* are highly expressed in mouse embryonic lung, human lung cancer cell lines, and in several mouse models of lung cancer.

A The two transcript isoforms of *Gata6* and *Nkx2-1* are differentially regulated during mouse lung development. Isoform-specific expression analysis was performed for both genes by qRT–PCR in mouse embryonic lung at the indicated day post-coitum (dpc) and in adult lung (Ad). Rel nor exp, relative expression normalized to *Tuba1a*. Error bars, standard error of the mean (s.e.m.); n = 3.

B The two transcript isoforms of *Gata6* and *Nkx2-1* are differentially regulated during lung cancer (LC) and show complementary expression. Isoform-specific gene expression analysis was performed for both genes by qRT–PCR in control lung tissue (Ctrl) and LC cell lines, A549, A427 (adenocarcinoma) and H322 (bronchoalveolar carcinoma). Rel nor exp, relative expression normalized to *TUBA1A*. Error bars, standard error of the mean (s.e.m.); n = 5.

C High expression of the Em isoform of G*ata6* and *Nkx2-1* in mouse models of LC. Isoform-specific expression analysis was performed in lungs from control mice (n = 3, Ctrl) and transgenic mouse models of LC, *Kras LA2* (K) and *SftpC*-driven *C-Raf BxB* (B), as well as xenograft LC models wherein A549 cells (A) were instilled intratracheally or LLC1 cells (L) were injected into the tail vein. The results from one control (Ctrl) and two experimental (T1, T2) mice are shown. Numbers indicate the Em/Ad expression ratio. Error bars, standard error of the mean (s.e.m.); n = 3.

D Exosomes are enriched in human lung cancer. Whole cell protein extracts from four control (Ctrl) and four lung cancer (LC) tissue samples were analyzed by Western blotting using antibodies specific for exosome markers (CD63 and TSG101) and beta actin (ACTB) as loading control.

E Isoform-specific expression analysis showed that Em and Ad transcripts of *GATA6* and *NKX2-1* are present in exosomes that were isolated from EBCs. Bacterial 16S/23S rRNA was added at the time point of exosome lysis and used as spike-in control. Data are represented as Em/Ad expression ratio. Each oval represents the mean of technical triplicates. The horizontal lines represent the means of biological replicates and the error bars the s.e.m..

© 2016 The Authors

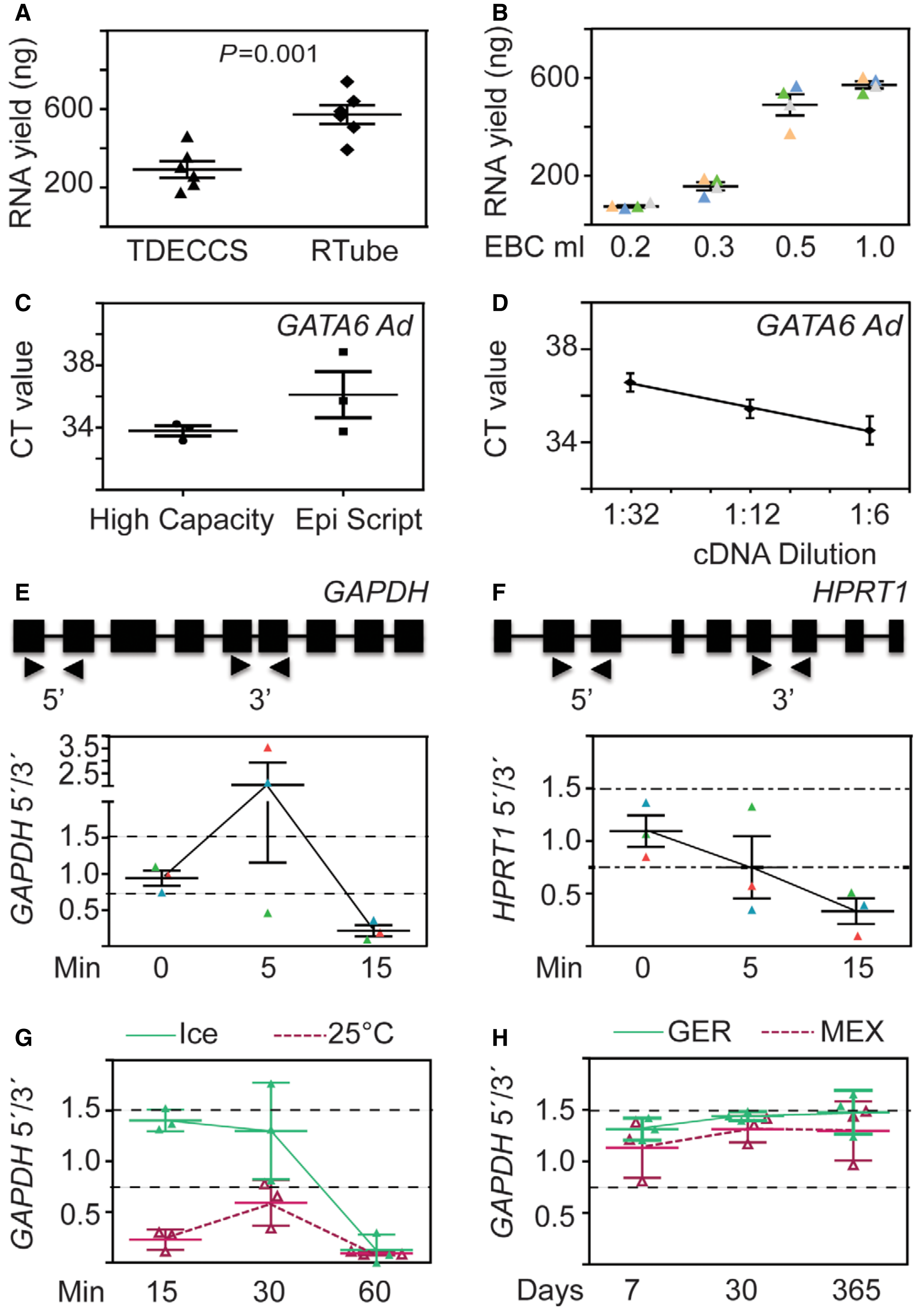
A
P=0.001
RNA yield (ng)
600
200
TDECCS
RTube
B
RNA yield (ng)
600
200
EBC ml
0.2
0.3
0.5
1.0
C
GATA6 Ad
CT value
38
34
High Capacity
Epi Script
D
GATA6 Ad
CT value
38
34
1:32
1:12
1:6
cDNA Dilution
E
GAPDH
5'
3'
GAPDH 5´/3´
3.5
2.5
1.5
1.0
0.5
Min
0
5
15
F
HPRT1
5'
3'
HPRT1 5´/3´
1.5
1.0
0.5
Min
0
5
15
G
Ice
25°C
GAPDH 5´/3´
1.5
1.0
0.5
Min
15
30
60
H
GER
MEX
GAPDH 5´/3´
1.5
1.0
0.5
Days
7
30
365

Figure EV3.

◀ **Figure EV3. Optimization of EBC-based expression analysis for LC diagnosis.**

A RTube is suitable for RNA isolation. Two main EBC collection devices, RTube and TurboDECCS, were compared for the total RNA yield (*y*-axis, ng) obtained using the QIAGEN RNeasy Micro kit and 500 μl EBC as starting material. Triangles and rhombuses are used to denote RNA yield of each individual sample using EBCs collected from the different EBC collection devices. Data are represented as mean ± s.e.m.; $n = 6$. *P*-values after one-way ANOVA.

B 500 μl of EBC is optimal for RNA isolation. Total RNA isolation with the RNeasy Micro kit was performed using 200, 350, 500, or 1,000 μl EBC as starting material. Data are represented as mean ± s.e.m.; $n = 4$.

C The High Capacity cDNA Reverse Transcriptase kit is more efficient than EpiScript Reverse Transcriptase. Two RT kits were tested for qRT–PCR-based analysis of *GATA6* Ad. CT values were plotted. Each dot represents the CT value for a technical triplicate. Data are represented as mean ± s.e.m.; $n = 3$.

D Serial dilution of cDNA template to determine the linear range of detection of *GATA6* Ad. cDNA from control EBC was serially diluted and used as template for qRT–PCR-based expression analysis of *GATA6* Ad. Each dot represents the CT value for a technical triplicate. Data are represented as mean ± s.e.m.; $n = 3$.

E, F Delayed snap-freezing of EBC after collection compromises mRNA integrity. Top, schematic representation of the precursor mRNAs from *GAPDH* (E) and *HPRT1* (F) showing exons (boxes), introns (lines), and location of primer pairs (arrowheads) used for qRT–PCR-based expression analysis. Bottom, EBCs were collected and incubated on ice for 0, 5, and 15 min prior to snap-freezing in liquid nitrogen. Expression of *GAPDH* and *HPRT1* was determined in the EBCs using the indicated primers, and the expression ratios (5′/3′) of each gene were calculated as indicators of mRNA integrity. RNA purified from EBCs with expression ratios of *GAPDH* and *HPRT1* between 0.75 and 1.5 (dashed lines) was considered as acceptable for further analysis. Data are represented as mean ± s.e.m.; $n = 3$. Each colored triangle represents one individual.

G EBCs should be thawed on ice, for a maximum of 15 min, before further processing. EBCs, that were stored at −80°C, were thawed on ice (green line) or 25°C (red dashed line) for 15, 30, and 60 min. *GAPDH* 5′/3′ expression ratios were determined as in (E) as indicators of mRNA integrity. Each triangle refers to the 5′/3′ expression ratio for *GAPDH* of one sample. Each sample was measured in triplicates and the mean was used for the calculation of the ratio.

H Long-term storage at −80°C or transportation on dry ice did not compromise mRNA integrity. EBCs were collected either in Germany (GER, green line) or in Mexico (MEX, red dashed line) and subsequently transported to Germany on dry ice. EBCs were stored at −80°C for 7, 30, or 365 days before they were thawed on ice and further processed in less than 15 min. *GAPDH* 5′/3′ expression ratios were determined as in (E) as indicators of mRNA integrity.

 © 2016 The Authors

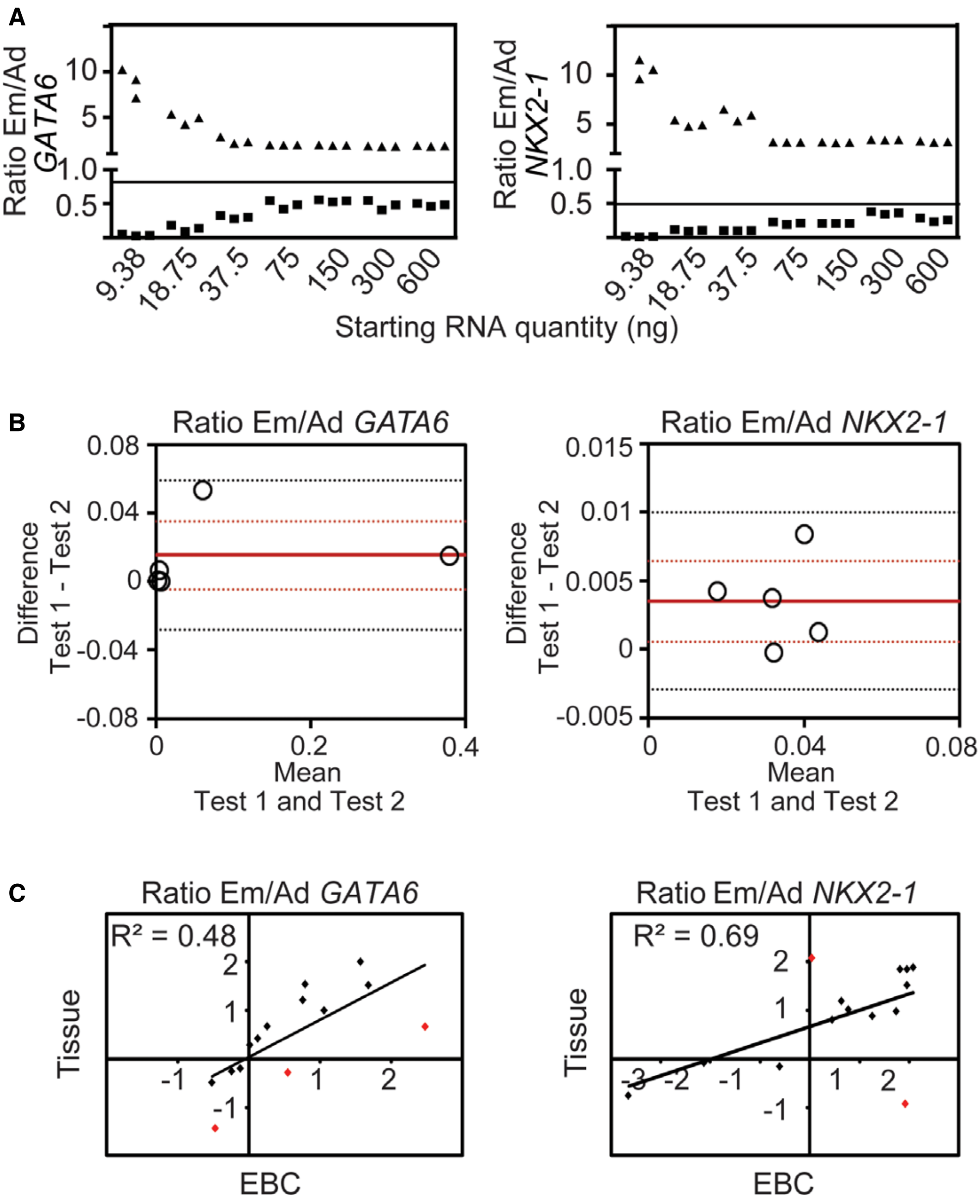

Figure EV4. Repeatability and reproducibility of isoform-specific expression analysis in EBCs.

A For reliable isoform-specific expression analysis in EBC, a minimum of 75 ng of starting RNA is required. Different amounts of RNA (*x*-axis, ng) were used for cDNA synthesis by RT reaction and subsequently isoform-specific expression analysis. The *GATA6* (left) and *NKX2-1* (right) Em/Ad ratio is plotted for both control (square) and lung cancer samples (triangle).

B Bland–Altman plots for Em/Ad expression ratios of *GATA6* (left) and *NKX2-1* (right) confirmed the repeatability of isoform-specific expression analysis in EBCs. Isoform-specific expression analysis of *GATA6* and *NKX2-1* was performed in two EBCs from the same individual (Test 1 and Test 2). Measurement of each isoform in each EBC was performed in triplicates. The Em/Ad ratios were calculated from the means. Five individuals were analyzed. Each dot represents one individual. The difference between Test 1 and Test 2 (*y*-axis) was plotted against their average (*x*-axis). Red solid line, mean of the differences; red dotted lines, 95% confidence interval of the mean of the differences; black dotted lines, upper and lower limits of agreement.

C Correlation between the values obtained from lung tissue sample and EBC for each patient. The *GATA6* (left) and *NKX2-1* (right) Em/Ad ratio for both lung tissue (*y*-axis) and EBC (*x*-axis) samples were log2-transformed and plotted. The linear regression was also plotted for both. Red dots, patients where the values from both sample types were significantly different.

© 2016 The Authors

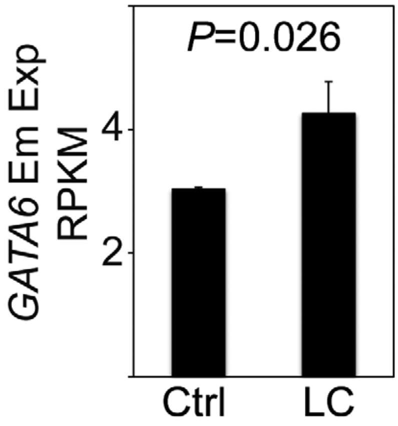

Figure EV5. Increased expression of *GATA6* Em in LC samples confirmed by RNA-sequencing data.
Analysis of RNA-sequencing data deposited at the Cancer Genome Atlas confirmed the increased expression of *GATA6* Em in LC samples. *GATA6* Em expression was analyzed using publically available RNA-seq data from the TCGA database of control (Ctrl) and LC samples. Analysis was performed specific for the exon unique to *GATA6* Em (exon 1 with the coordinates chr18:19749416-19749643 from the human genome hg19). The reads per kilobase of exon model per million mapped reads (RPKM) are plotted. Data are represented as mean $\pm$ s.e.m.; $n = 5$. P-value after one-way ANOVA.

© 2016 The Authors
