# Supplementary material for: Non‐invasive lung cancer diagnosis by detection of GATA6 and NKX2‐1 isoforms in exhaled breath condensate
Source: EMBO Mol Med. 2016 Nov 7;8(12):1380–9. doi: 10.15252/emmm.201606382 (PMC5167131; doi:10.15252/emmm.201606382)
Supplement: Supplementary file 3 — Tables EV1–EV4 [file EMMM-8-1380-s003.docx]

**EXPANDED VIEW TABLES**

**Table EV1: Five-number summaries of box plots and results of statistical significance tests.**

|  | **FFPE tissue samples** | | | | | | | | | | | | | | | | | | | | **Exhaled Breath Condensates** | | | | | | | |
| --- | --- | --- | --- | --- | --- | --- | --- | --- | --- | --- | --- | --- | --- | --- | --- | --- | --- | --- | --- | --- | --- | --- | --- | --- | --- | --- | --- | --- |
|  | **Fig 1B** | | | | **Fig 1C** | | | | | | | | | | | | | | | | **Fig 2A** | | | | **Fig 2B** | | **Fig 2C** | |
|  | **Ratio Em/Ad** | | | | **Ratio Em/Ad *GATA6*** | | | | | | | | **Ratio Em/Ad *NKX2-1*** | | | | | | | | **Ratio Em/Ad** | | | | **LC Score** | | **LC Score** | |
|  | *GATA6* | | *NKX2-1* | | GER | | MEX | | Female | | Male | | GER | | MEX | | Female | | Male | | *GATA6* | | *NKX2-1* | | Training | | Validation | |
|  | Ctrl | LC | Ctrl | LC | Ctrl | LC | Ctrl | LC | Ctrl | LC | Ctrl | LC | Ctrl | LC | Ctrl | LC | Ctrl | LC | Ctrl | LC | Ctrl | LC | Ctrl | LC | Ctrl | LC | Ctrl | LC |
| *n* | 61 | 51 | 61 | 51 | 54 | 38 | 7 | 13 | 9 | 23 | 8 | 16 | 54 | 38 | 7 | 13 | 9 | 23 | 8 | 16 | 65 | 48 | 65 | 48 | 65 | 48 | 78 | 60 |
| min | 0.003 | 0.54 | 0.003 | 0.20 | 0.003 | 0.54 | 0.19 | 0.70 | 0.03 | 0.72 | 0.03 | 0.91 | 0.003 | 0.20 | 0.07 | 0.48 | 0.007 | 0.20 | 0.008 | 1.10 | 0.0004 | 0.04 | 0.01 | 0.37 | -10.29 | -0.83 | -9.15 | -0.05 |
| Q1 | 0.01 | 1.59 | 0.07 | 1.24 | 0.01 | 1.84 | 0.35 | 0.93 | 0.05 | 1.60 | 0.14 | 1.40 | 0.05 | 1.16 | 0.24 | 1.29 | 0.18 | 1.38 | 0.04 | 1.39 | 0.06 | 0.87 | 0.02 | 0.71 | -5.81 | 1.00 | -5.67 | 2.19 |
| Median | 0.15 | 2.25 | 0.18 | 1.62 | 0.06 | 2.56 | 0.45 | 1.09 | 0.45 | 2.20 | 0.44 | 2.50 | 0.17 | 1.59 | 0.36 | 1.80 | 0.30 | 1.70 | 0.31 | 2.07 | 0.15 | 1.32 | 0.23 | 1.13 | -2.66 | 1.56 | -4.07 | 2.99 |
| Q3 | 0.81 | 3.32 | 0.42 | 2.84 | 0.82 | 3.65 | 0.51 | 1.99 | 0.82 | 2.84 | 0.80 | 4.00 | 0.47 | 2.69 | 0.37 | 3.69 | 0.36 | 2.55 | 0.49 | 3.71 | 0.31 | 2.05 | 0.35 | 2.00 | -1.26 | 2.59 | -1.54 | 4.37 |
| max | 1.40 | 5.55 | 0.88 | 4.24 | 2.01 | 5.55 | 0.55 | 3.23 | 0.89 | 4.34 | 0.82 | 4.64 | 0.88 | 3.92 | 0.48 | 4.24 | 0.62 | 4.24 | 0.50 | 6.96 | 0.60 | 3.32 | 0.62 | 3.68 | 3.12 | 4.09 | 2.33 | 6.75 |
| χ^2^ | 66.2 | | 75.8 | | 57.6 | | 11.9 | | 16.4 | | 15.4 | | 60.4 | | 12.4 | | 16.4 | | 16.4 | | 68.3 | | 72.6 | | 75.3 | | 97.6 | |
| df | 1 | | 1 | | 1 | | 1 | | 1 | | 1 | | 1 | | 1 | | 1 | | 1 | | 1 | | 1 | | 1 | | 1 | |
| *P* | 4.1E-16 | | 3.1E-18 | | 3.2E-14 | | 5.7E-04 | | 5.2E-05 | | 8.9E-05 | | 7.7E-15 | | 6.0E-04 | | 5.2E-05 | | 5.2E-05 | | 2.2E-16 | | 1.6E-17 | | 4.0E-18 | | 4.9E-23 | |

Five number summaries of the box plots presented in the indicated Figures. Minimum (min) and maximum (max) values; first (Q1) and third (Q3) quartile. Kruskal-Wallis test was performed to determine the statistical significance of the difference between control (Ctrl) and lung cancer (LC) samples. Chi-squared (χ2); degree of freedom (df) and *P*-values are presented. FFPE, formalin-fixed and paraffin-embedded tissue samples; Samples collected in Germany (GER) and Mexico (MEX).

**Table EV2: Five-number summaries of box plots and results of statistical significance tests.**

|  | **FFPE tissue samples** | | | | | | | | | | **Exhaled Breath Condensates** | | | | | | | |
| --- | --- | --- | --- | --- | --- | --- | --- | --- | --- | --- | --- | --- | --- | --- | --- | --- | --- | --- |
|  | **Fig 1D** | | | | | | | | | | **Fig 3B** | | | **Fig 3C** | | | | |
|  | **Ratio Em/Ad *GATA6*** | | | | | **Ratio Em/Ad *NKX2-1*** | | | | | **LC score** | | | **LC score** | | | | |
|  | Ctrl | LC I | LC II | LC III | LC IV | Ctrl | LC I | LC II | LC III | LC IV | Ctrl | NSCLC | SCLC | Ctrl | LC I | LC II | LC III | LC IV |
| *n* | 61 | 12 | 14 | 5 | 8 | 61 | 12 | 14 | 5 | 8 | 143 | 58 | 9 | 143 | 9 | 11 | 29 | 12 |
| min | 0.003 | 1.13 | 1.73 | 1.01 | 0.70 | 0.003 | 1.13 | 0.20 | 3.78 | 0.48 | -10.29 | 0.12 | 0.24 | -10.29 | 0.24 | -0.83 | -0.05 | 0.20 |
| Q1 | 0.01 | 1.93 | 2.62 | 1.53 | 0.78 | 0.07 | 1.46 | 1.29 | 3.78 | 1.20 | -5.66 | 1.46 | 0.80 | -5.67 | 1.08 | 1.23 | 1.98 | 0.73 |
| Median | 0.15 | 2.23 | 3.13 | 1.65 | 0.92 | 0.18 | 1.67 | 2.31 | 3.85 | 1.48 | -3.34 | 2.22 | 1.32 | -3.66 | 1.52 | 2.89 | 2.68 | 1.39 |
| Q3 | 0.81 | 2.69 | 3.91 | 1.99 | 1.06 | 0.42 | 2.19 | 3.74 | 3.89 | 1.90 | -1.20 | 3.05 | 3.08 | -1.27 | 2.39 | 4.29 | 3.54 | 2.50 |
| max | 1.38 | 3.23 | 4.42 | 1.99 | 1.09 | 0.88 | 2.41 | 6.96 | 3.89 | 1.99 | 3.12 | 4.78 | 4.91 | 3.12 | 4.32 | 5.12 | 4.78 | 4.75 |
| df |  |  | 4 |  |  |  |  | 4 |  |  |  | 3 |  |  |  | 4 |  |  |
| Ctrl-I | 4.6E-10 |  |  |  |  | 6.3E-03 |  |  |  |  |  |  |  | 1.0E-06 |  |  |  |  |
| Ctrl-II | 4.6E-10 |  |  |  |  | 7.7E-06 |  |  |  |  |  |  |  | 1.0E-06 |  |  |  |  |
| Ctrl-III | 8.7E-06 |  |  |  |  | 9.4E-05 |  |  |  |  |  |  |  | 1.0E-06 |  |  |  |  |
| Ctrl-IV | 4.0E-03 |  |  |  |  | 2.5E-05 |  |  |  |  |  |  |  | 1.0E-06 |  |  |  |  |
| I-II |  | 2.7E-03 |  |  |  |  | 0.76 |  |  |  |  |  |  |  | 0.94 |  |  |  |
| I-III |  | 0.92 |  |  |  |  | 0.26 |  |  |  |  |  |  |  | 0.93 |  |  |  |
| I-IV |  | 0.001 |  |  |  |  | 0.43 |  |  |  |  |  |  |  | 0.99 |  |  |  |
| II-III |  |  | 0.02 |  |  |  |  | 0.77 |  |  |  |  |  |  |  | 0.99 |  |  |
| II-IV |  |  | 1.1E-08 |  |  |  |  | 0.95 |  |  |  |  |  |  |  | 0.88 |  |  |
| III-IV |  |  |  | 0.12 |  |  |  |  | 0.98 |  |  |  |  |  |  |  | 0.83 |  |
| Ctrl-NSCLC |  |  |  |  |  |  |  |  |  |  | 1.0E-06 |  |  |  |  |  |  |  |
| Ctrl-SCLC |  |  |  |  |  |  |  |  |  |  | 1.0E-06 |  |  |  |  |  |  |  |
| NSCLC-SCLC |  |  |  |  |  |  |  |  |  |  |  | 0.97 |  |  |  |  |  |  |

Five number summaries of the box plots presented in the indicated Figures. Minimum (min) and maximum (max) values; first (Q1) and third (Q3) quartile. Tukey´s HSD test after one-way ANOVA were performed to determine the statistical significance of the difference between the groups. Degree of freedom (df) and *P*-values of the comparison between the indicated groups are presented. FFPE, formalin-fixed and paraffin-embedded tissue samples; Ctrl, control; LC, lung cancer. LC I to LC IV, samples were staged according to the TNM Classification (UICC, 7^th^ edition). NSCLC. non-small cell lung cancer; SCLC. small cell lung cancer.

**Table EV3: Five-number summaries of box plots and results of statistical significance tests.**

|  | **Exhaled Breath Condensates** | | | | | |
| --- | --- | --- | --- | --- | --- | --- |
|  | **Fig 3A** | | | | | |
|  | **LC score NS** | | **LC score PS** | | **LC score CS** | |
|  | Ctrl | LC | Ctrl | LC | Ctrl | LC |
| *n* | 24 | 9 | 2 | 34 | 6 | 13 |
| min | -2.34 | -0.05 | -2.45 | 0.12 | -2.97 | 0.84 |
| Q1 | -1.85 | 1.85 | -2.45 | 2.01 | -2.97 | 2.03 |
| Median | -1.24 | 3.32 | -1.34 | 2.99 | -2.58 | 2.25 |
| Q3 | -0.79 | 3.64 | -0.22 | 4.51 | -1.73 | 3.00 |
| max | 0.28 | 4.34 | -0.22 | 6.57 | -1.27 | 3.21 |
| df Smoking (s) |  |  |  |  |  | 1 |
| df Disease (d) |  |  |  |  |  | 2 |
| df s*d |  |  |  |  |  | 2 |
| Ctrl:CS-LC:CS |  |  |  |  | 1.0E-05 |  |
| Ctrl:NS-Ctrl:CS | 0.13 |  |  |  |  |  |
| Ctrl:CS-LC:NS |  |  |  |  | 1.0E-05 |  |
| Ctrl:PS-Ctrl:CS |  |  | 0.77 |  |  |  |
| Ctrl:CS-LC:PS |  |  |  |  | 1.0E-07 |  |
| Ctrl:NS-LC:CS | 1.0E-06 |  |  |  |  |  |
| LC:NS-LC:CS | 0.99 |  |  |  |  |  |
| Ctrl:PS-LC:CS |  |  | 1.6E-03 |  |  |  |
| LC:PS-LC:CS |  |  | 0.60 |  |  |  |
| Ctrl:NS-LC:NS | 1.0E-05 |  |  |  |  |  |
| Ctrl:NS-Ctrl:PS | 0.99 |  |  |  |  |  |
| Ctrl:NS-LC:PS | 1.0E-06 |  |  |  |  |  |
| Ctrl:PS-LC:NS |  |  | 1.4E-03 |  |  |  |
| LC:NS-LC:PS | 0.90 |  |  |  |  |  |
| Ctrl:PS-LC:PS |  |  | 6.3E-04 |  |  |  |

Five number summaries of the box plots presented in the indicated Figures. Minimum (min) and maximum (max) values; first (Q1) and third (Q3) quartile. Tukey´s HSD test after multivariate ANOVA were performed to determine the statistical significance of the difference between the groups. Degree of freedom (df) and *P*-values of the comparison between the indicated groups are presented. Ctrl, control; LC, lung cancer; NS, never smoker; PS, previous smoker; CS, current smoker.

**Table EV4: LC performance validation for different subsampling of the control and LC patients.**

| **Performace** | **Validation set** | **Ctrl w/o IPF/COPD** | **Smokers** | **LC w/o recurrent** |
| --- | --- | --- | --- | --- |
| PPV | 0.88 | 0.94 | 1.00 | 0.87 |
| NPV | 0.99 | 0.96 | 1.00 | 0.99 |
| TPR (sensitivity) | 0.98 | 0.98 | 1.00 | 0.98 |
| TNR (specificity) | 0.90 | 0.87 | 1.00 | 0.90 |

Positive predicted value (PPV), negative predicted value (NPV), true positive rate (TPR), true negative rate (TNR). **1**) Validation set, control (healthy, IPF, COPD). **2**) Samples from validation set, tacking as control only the healthy donors. **3**) Taking the previous smoker (PS) and current smoker (CS) from both groups of the validation set. **4**) Samples from validation set, excluding recurrent cases and tacking only incident cases in the LC group
