# Supplementary material for: Non‐invasive lung cancer diagnosis by detection of GATA6 and NKX2‐1 isoforms in exhaled breath condensate
Source: EMBO Mol Med. 2016 Nov 7;8(12):1380–9. doi: 10.15252/emmm.201606382 (PMC5167131; doi:10.15252/emmm.201606382)
Supplement: Supplementary file 4 — Dataset EV1 [file EMMM-8-1380-s004.zip › Dataset_EV1/Read_me.rtf]

Two files are provided for reproducing the results presented in the main figures and in the tables: an R Markdown file (GATA6_NKX2_1_EBC.Rmd) together with a file containing the raw data (Raw_data.csv) as Dataset EV1. (I) Both files have to be located into the folder, in which the output of the R Markdown should be saved. To run the R Markdown file, R version ≥2.15.0 and R Studio are required. (II) After opening the R Markdown file with R Studio, the Knitr option should be selected. (IV) Using the raw data, the R Markdown will generate an html file containing all the plots from the main figures. In addition, several txt files containing the data from Table 2 and Tables EV1-4 will be also generated.
